# Bimodal Control of Dendritic and Axonal Growth by the Dual Leucine Zipper Kinase Pathway

**DOI:** 10.1371/journal.pbio.1001572

**Published:** 2013-06-04

**Authors:** Xin Wang, Jung Hwan Kim, Mouna Bazzi, Sara Robinson, Catherine A. Collins, Bing Ye

**Affiliations:** 1 Life Sciences Institute and Department of Cell and Developmental Biology, University of Michigan, Ann Arbor, Michigan, United States of America; 2 Department of Molecular, Cellular and Developmental Biology, University of Michigan, Ann Arbor, Michigan, United States of America; The Scripps Research Institute, United States of America

## Abstract

Knowledge of the molecular and genetic mechanisms underlying the separation of dendritic and axonal compartments is not only crucial for understanding the assembly of neural circuits, but also for developing strategies to correct defective dendrites or axons in diseases with subcellular precision. Previous studies have uncovered regulators dedicated to either dendritic or axonal growth. Here we investigate a novel regulatory mechanism that differentially directs dendritic and axonal growth within the same neuron in vivo. We find that the dual leucine zipper kinase (DLK) signaling pathway in *Drosophila*, which consists of Highwire and Wallenda and controls axonal growth, regeneration, and degeneration, is also involved in dendritic growth in vivo. Highwire, an evolutionarily conserved E3 ubiquitin ligase, restrains axonal growth but acts as a positive regulator for dendritic growth in class IV dendritic arborization neurons in the larva. While both the axonal and dendritic functions of *highwire* require the DLK kinase Wallenda, these two functions diverge through two downstream transcription factors, Fos and Knot, which mediate the axonal and dendritic regulation, respectively. This study not only reveals a previously unknown function of the conserved DLK pathway in controlling dendrite development, but also provides a novel paradigm for understanding how neuronal compartmentalization and the diversity of neuronal morphology are achieved.

## Abstract 2

The dual leucine zipper kinase pathway acts on two separate transcriptional programs to dichotomously control dendritic and axonal growth.

## Author Summary

Dendrites and axons are the input and output compartments of a neuron, respectively. Understanding how dendrites and axons are separated during neuronal development may help in developing strategies to correct defective neurons in neurological disorders and injuries. We show here that an evolutionarily conserved molecular pathway dichotomously controls dendritic and axonal growth. A key molecule in this pathway, dual leucine zipper kinase (DLK), suppresses dendritic growth but promotes axonal growth. While DLK is known to be a key regulator of axon growth and regeneration, this study reveals its roles in dendritic growth for the first time. In addition, we find that the DLK pathway diverges through two separate downstream programs that control the expression of other genes. These insights can help target this pathway to specifically promote axon regeneration without affecting dendritic growth. Overall, these results help provide a new framework for understanding neuronal compartmentalization and the diversity of neuronal morphology.

## Introduction

The separation of the dendritic and axonal compartments in neurons is prerequisite to the function of neural circuits. Although the difference between dendrites and axons is a cornerstone of modern neuroscience, as theorized in the “neuron doctrine” by Ramon y Cajal [1], our molecular understanding of how neuronal compartmentalization is achieved remains limited. This knowledge, however, is crucial for understanding the assembly of neural circuits. Moreover, it is needed to develop strategies that will correct defective dendrites or axons with subcellular precision, and to alter the wiring of neural circuits in animal models in order to interrogate the functions of the nervous system.

Previous studies have demonstrated the existence of regulators dedicated to dendrite or axon growth in the same neuron, referred to as “dedicated mechanisms” herein. For instance, the transcription complex, p300–SnoN, specifically promotes axon growth in the cerebellar granule neurons [2]. In contrast, transcription factor NeuroD is dedicated to dendritic growth in mammalian cerebellar granule neurons [3]. Likewise, bone morphogenetic protein 7 (BMP7) specifically promotes dendritic growth in several types of neurons in culture [4],[5]. In *Drosophila*, the transcription factor Dendritic arbor reduction 1 (Dar1) promotes dendritic, but not axonal, growth [6]. In addition, dendritic and axonal growth exhibit differences in their dependence on the secretory pathway [7].

Besides the dedicated mechanisms, another way to differentiate dendritic and axonal growth is through bimodal regulators that control dendritic and axonal growth in opposite directions [8]–[11]. Different from the dedicated mechanisms, the bimodal mechanisms may coordinate growth of the two neuronal compartments. However, how the function of a molecule or signaling pathway diverges into distinct dendritic and axonal regulations is poorly known.

In this study we report that the dual leucine zipper kinase (DLK) signaling pathway is a novel bimodal regulator for dendritic and axonal growth in vivo. The core players in the DLK signaling pathway are the DLK and the Pam/Highwire/RPM-1 (PHR) family of E3 ubiquitin ligases that suppress DLK expression. The PHR-DLK signaling module plays an important role in axon development, as demonstrated by studies in *C. elegans* [12]–[14], *Drosophila* [15]–[17], zebrafish [18],[19], and mammals [20]–[22]. Loss of the *Drosophila* homologue of DLK-1, Wallenda (Wnd), suppresses the axonal overgrowth caused by loss of the PHR protein Highwire (Hiw) [15],[17]. Consistently, overexpression of Wnd promotes axonal growth of motoneurons in *Drosophila* larvae [17]. In *Drosophila* adult mushroom body neurons, Hiw-Wnd pathway is required for the segregation of axon branches in response to guidance cues [23]. In addition to the roles in axon development, recent studies have discovered a conserved function of the DLK pathway in axon regeneration [24]–[28] and degeneration in several species [29]–[32]. Although these exciting findings have established critical roles for the DLK pathway in axon development, regeneration, and degeneration, whether the DLK pathway regulates dendrites remains unknown.

Here we show that the DLK pathway directs the growth of axons and dendrites in opposite directions in the class IV dendritic arborization (C4da) neurons in *Drosophila*. By inhibiting Wnd functions, Hiw restricts axonal growth but promotes dendritic growth. The opposite effects of the Hiw-Wnd pathway on axons and dendrites are achieved through two distinct transcription factors: Fos, which mediates the regulation of axonal growth, and Knot (Kn), which mediates the regulation of dendritic growth. Collectively, these results demonstrate that a single signaling pathway can differentiate dendritic and axonal growth through two independent transcriptional programs.

## Results

### *hiw* Plays a Dichotomous Role in Differentiating Dendrite and Axon Growth

All functional studies of the PHR-DLK pathway in neurons have so far focused on axons. We first set out to determine whether the PHR gene *hiw* is involved in dendrite development using *Drosophila* as a model system.

The C4da neurons in *Drosophila* larva are a well-established in vivo system for studying the molecular mechanisms of dendrite and axon development. The dendrites and axons of these neurons are distinguishable from each other at both molecular and organelle levels in a way that resembles mammalian neurons [33]. Moreover, these neurons are amenable to single-cell genetic manipulations [33],[34], which is important for comparing dendritic and axonal development in vivo. In each hemi-segment of a larva, there are three C4da neurons (ddaC, v'ada, and vdaB), whose cell bodies are located respectively in the dorsal, lateral, and ventral parts of the body wall. The axons of the three C4da neurons extend to the ventral nerve cord (VNC) where the terminals form a ladder structure (Figure S1A). At single-cell resolution, the axon terminal of each C4da neuron consists of an anterior projection that extends within one segment length. ddaC and vdaB neurons also extend a contra-lateral branch and sometimes a posterior branch (Figure S1A′) [35]. Collectively, the axon terminals of the three C4da neurons form a fascicle that connects two adjacent neuropils (Figure S1A′).

**Figure 1:**
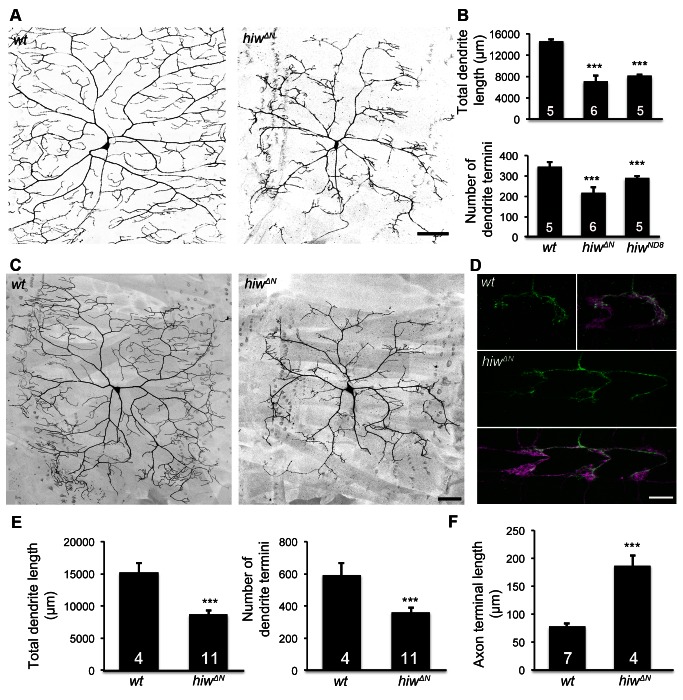
Hiw differentially regulates dendrite and axon growth in C4da neurons. (A) Dendrites of the C4da neuron ddaC in *hiw^ΔN^* homozygous mutant larvae are reduced, as compared to *wild-type* (*wt*). C4da neurons were labeled by the C4da marker *ppk-CD4::tdTomato*. Scale bar, 100 µm. (B) Bar charts showing the quantification of total dendrite length (top), number of dendrite termini (bottom) of ddaC in *wt*, *hiw^ΔN^*, and *hiw^ND8^* larvae. Sample numbers are shown in the bars of the bar charts throughout this article. (C–D) *hiw* mutant MARCM clones exhibit impaired dendritic growth and overgrowth of axon terminals. (C) Representative dendrites of *wt* and *hiw^ΔN^* mutant ddaC neurons. Scale bar, 50 µm. (D) Representative axon terminals of a single *wt* ddaC and a single *hiw^ΔN^* mutant ddaC. The axon terminals of wild-type ddaC clones (green) extend within one segment length of the C4da neuropil (magenta) labeled by *ppk-CD4::tdTomato*. The axon terminals of *hiw^ΔN^* mutant clones (green) expand over multiple segment lengths of the C4da neuropil (magenta). Scale bar, 10 µm. (E) Quantification of total dendrite length (left) and number of dendrite termini (right) of *wt* and *hiw^ΔN^* MARCM clones. (F) Quantification of axon terminal length of *wt* and *hiw^ΔN^* MARCM clones.

To examine the role of *hiw* in dendritic development, we labeled the C4da neurons in *hiw* mutant larvae using a C4da-specific marker, *ppk-CD4::tdTomato* [34],[36]. We found that dendritic growth was dramatically reduced in the null allele *hiw^ΔN^* and, to a lesser extent, in the hypomorphic *hiw^ND8^* mutants (Figure 1A and B). Both total length and number of termini of dendrites were significantly reduced in *hiw^ΔN^* and *hiw^ND8^* mutants (Figure 1B).

Consistent with the known function of *hiw* in suppressing axonal growth [15],[16], *hiw* mutations led to exuberant growth of axon terminals in C4da neurons. In *hiw* mutant larvae, thickened connective fascicles were observed in the C4da neuropil ladder (Figure S1B). In wild-type larvae, there was no hemi-segment that contained more than three longitudinal connectives between the axon entry point of abdominal segment 5 (A5) and that of A6 (Figure S1A′,B, and D). In contrast, 100% of *hiw* mutant C4da neuropils exhibited more than three connectives (Figure S1B and D), which could either arise from an increased number of axon branches from neurons in the same segment or from overextended axons that normally remain in other segments.

Our further analysis showed that the effects of *hiw* mutations on dendritic and axonal growth are not a result of defective dendrite and axon identities. The axon-specific marker, Kinesin-β-galactosidase [37],[38], remained exclusively localized to the axons of C4da neurons that were mutant for *hiw* (Figure S2A). Furthermore, the initial growth and pathfinding of axons to the VNC or the extension of minor dendritic processes remained unaltered in embryos devoid of both maternal and zygotic *hiw* (Figure S2B). Thus, *hiw* appears to be dispensable for early development, including the initial specification of axon and dendrite. Taken together, these results suggest that *hiw* plays a dichotomous role in differentiating dendrite and axon growth after their identities have been specified.

### Hiw Regulates Dendritic and Axonal Growth in a Cell-Autonomous Manner

Previous studies of axon development have discovered both cell-autonomous [16],[17] and non-cell-autonomous roles of *hiw* [23]. To determine whether *hiw* functions cell-autonomously in C4da neurons and to examine the axon and dendrite defects at single-neuron resolution, we generated *hiw* mutant neurons with the Mosaic Analysis with a Repressible Cell Marker (MARCM) technique [39]. Consistent with the reduced dendritic growth in *hiw* mutant larvae, we observed a reduction of high-order dendritic branches in *hiw* loss-of-function mutant neurons (Figure 1C). Moreover, fewer dendritic branches arrived at the segment border as compared to wild-type. *hiw* mutations caused a 43% reduction in total dendrite length and 40% reduction of the number of dendrite termini (Figure 1E). In contrast to their dendritic defects, *hiw* mutations resulted in a 2.4-fold increase of axon terminal length (Figure 1D and F) as compared to wild-type. The axon terminals of *hiw* mutant neurons typically spanned multiple segments, whereas the vast majority of wild-type C4da neurons extended axonal branches between their own segments and the anterior neighboring segments (Figure 1D). Noticeably, although the axon terminals of *hiw* mutant neurons grew exuberantly, they preserved normal guidance within the C4da neuropil tracts.

In agreement with the MARCM results, overexpressing Hiw in C4da neurons rescued both dendritic and axonal defects in *hiw* mutant larvae to a level comparable to wild-type (Figure S3), further confirming that the loss of *hiw* in C4da neurons is responsible for the dendritic and axonal defects. Overexpression of Hiw alone did not significantly alter axonal or dendritic growth (Figure S3), suggesting that *hiw* is necessary but insufficient to instruct dendritic growth and restrict axon growth. Taken together, these results demonstrate that Hiw functions as a cell-intrinsic bimodal regulator of dendritic and axonal growth in C4da neurons.

### Wnd Mediates the Functions of Hiw on Both Axonal and Dendritic Growth

**Figure 2:**
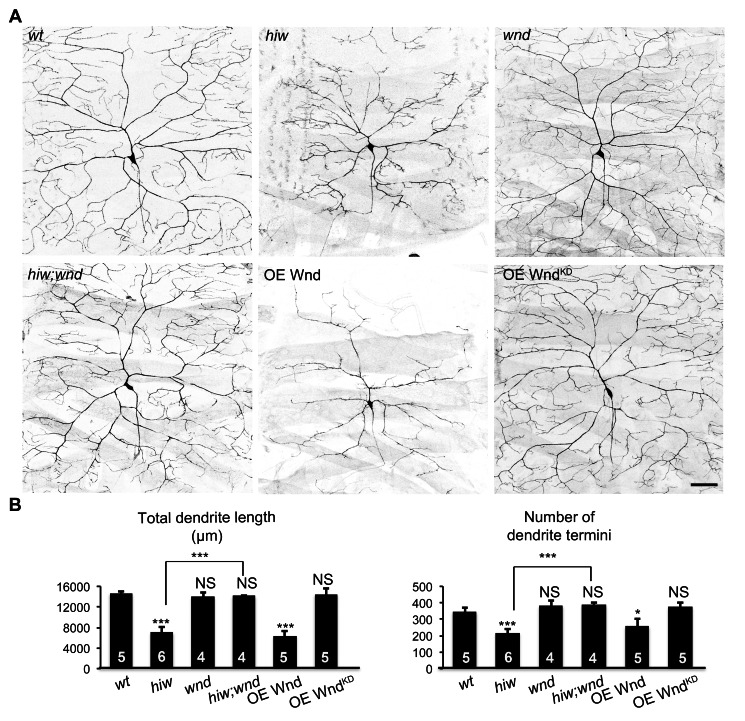
Wnd mediates the functions of Hiw on dendritic growth. (A) Loss of *wnd* blocks dendrite reduction in *hiw* mutants, and ectopic Wnd restrains dendritic growth. Shown are representative dendrites of ddaC neurons, labeled by *ppk-CD4::tdTomato*, of the following genotypes: (1) *wt*; (2) *hiw^ΔN^* homozygotes (*hiw*); (3) *wnd^1^/wnd^3^*(*wnd*); (4) *hiw^ΔN^*; *wnd^1^/wnd^3^* double mutants (*hiw*; *wnd*); (5) overexpressing Wnd by *ppkGal4* (OE Wnd); (6) overexpressing a kinase dead form (K188A) of Wnd by *ppkGal4* (OE Wnd^KD)^. Scale bar, 50 µm. (B) Bar charts showing the quantification of total dendrite length (left) and number of dendrite termini (right). Samples of *wt* and *hiw^ΔN^* that are used for statistical analysis are the same as those in Figure 1.

Two parallel downstream pathways are known to mediate axon overgrowth induced by loss of PHR proteins. First, the PHR orthologs in *C. elegans* (*rpm-*1) and *Drosophila* (*hiw*) suppresses the worm *dlk-1* and the fly *DLK wallenda* (*wnd*), respectively, to restrain axonal growth in motoneurons [14],[17]. Second, the worm *rpm-1* regulates a trafficking pathway that consists of the Rab guanine nucleotide exchange factor (GEF) GLO-4 and the Rab GTPase GLO-1, which restrict axon extension in mechanosensory neurons and synaptogenesis in motoneurons [40]. In order to delineate the mechanism underlying the bimodal control of dendritic and axonal growth by *hiw*, we tested the involvement of these two pathways in axon and dendrite growth in C4da neurons. While *wnd* loss-of-function mutations on their own did not alter axonal (Figure S1C–D) or dendritic morphology (Figure 2), they completely suppressed both axonal and dendritic defects caused by *hiw* mutations (Figure S1C–D and Figure 2). These observations suggest that *wnd* acts downstream of *hiw* to promote axonal growth and inhibit dendritic growth.

Consistent with this model, overexpression of Wnd in C4da neurons induced extensive axon terminal overgrowth and profoundly reduced dendritic branching in C4da neurons (Figure S1C–D and Figure 2). In contrast, overexpression of a kinase-dead (KD) form of Wnd resulted in morphologically normal C4da neurons (Figure S1C–D and Figure 2). Hence, increased expression of the Wnd kinase is sufficient to inhibit dendritic growth and promote axonal growth.

We also examined the potential involvement of the Rab trafficking pathway by testing *Drosophila* homologs of *glo-4* and *glo-1* in axon and dendrite development in C4da neurons. In *C. elegans*, *glo-4* mutants exhibited axon overextension similar to that in *rpm-1* mutants [40]. Overexpressing the Rab GTPase Glo-1, which is activated by Glo-4, partially rescued axon termination defects in *rpm-1* mutants [40]. The *Drosophila* homologs of *glo-4* and *glo-1* are *claret* (*ca*) and *lightoid* (*ltd*), respectively [41]. The *ca* mutant MARCM clones devoid of maternal contribution exhibited axons and dendrites that were indistinguishable from wild-type clones (Figure S4A–D). In addition, overexpressing Ltd failed to rescue either axon or dendrite defects in *hiw* mutants (Figure S4E–H). These results suggest that *Drosophila* C4da neurons use the DLK (Wnd) pathway, rather than the Ca-Ltd vesicle trafficking pathway, to mediate *hiw* function in axonal and dendritic growth.

### The Fos Transcription Factor Mediates the Hiw-Wnd Control of Axonal Growth

How might the Hiw-Wnd pathway control axonal and dendritic growth differently in the same neurons? In *Drosophila* motoneurons, the Hiw-Wnd pathway requires the transcription factor Fos [17]. Fos is phosphorylated by Bsk (JNK) [42], which positions it as the downstream kinase of the Wnd-Hep7-JNK kinase cascade [17]. Overexpressing a dominant negative form of Fos partially suppresses axonal overgrowth at the NMJ of *hiw* mutants [17]. Because of this, we decided to examine whether Fos is required by Wnd to promote axonal growth in C4da neurons.

**Figure 3:**
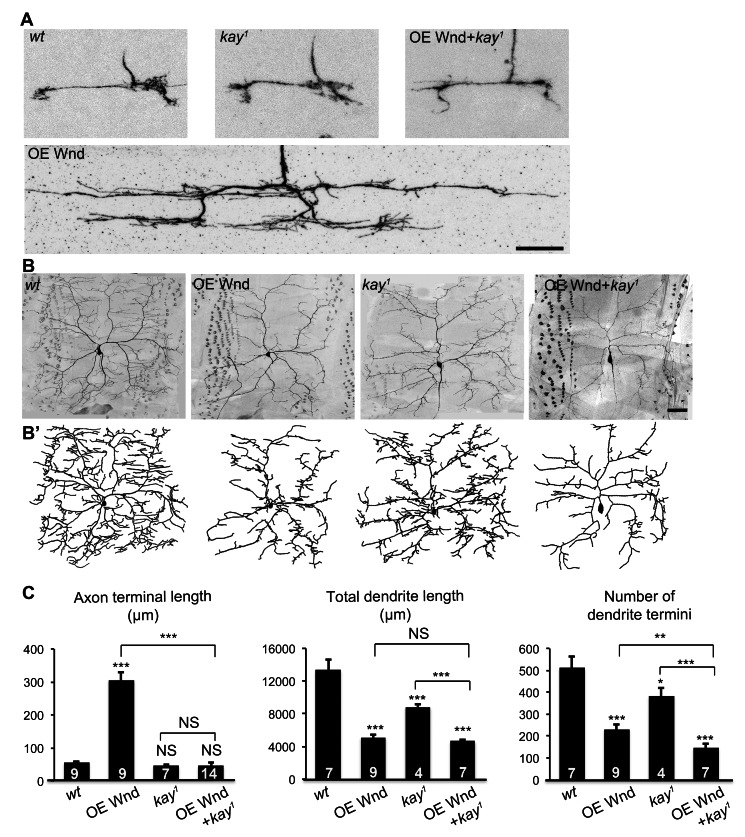
Transcription factor Fos specifically mediates axonal overgrowth induced by Wnd. (A) Loss of the *Drosophila fos*, *kay*, blocks axonal overgrowth caused by Wnd overexpression. Shown are representative axon terminals of ddaC MARCM clones of following genotypes: (1) *wt*; (2) overexpressing Wnd with MARCM (OE Wnd); (3) *kay^1^*; (4) overexpressing Wnd in *kay^1^* genetic background with MARCM (OE Wnd+*kay^1^*). Scale bar, 10 µm. (B–B′) *kay^1^* impairs dendritic growth in *wt* genetic background and exacerbates the dendritic reduction caused by Wnd overexpression. Shown are representative dendrites (B) and tracings (B′) of ddaC MARCM clones of indicated genotypes. Scale bar, 50 µm. (C) Bar charts showing the quantification of axon terminal length (left), total dendrite length (middle), and number of dendrite termini (right).

To test the role of Fos with loss-of-function mutants, and to bypass lethality caused by *fos* null mutations *kay^1^* [43],[44], we generated *kay^1^* MARCM clones in the presence or absence of a *UAS-Wnd* transgene that overexpresses Wnd (OE Wnd). *kay^1^* alone did not alter axonal growth (Figure 3A), but completely suppressed the axon overextension caused by Wnd overexpression (Figure 3A and C), which suggests that *fos* is required for Wnd-induced axonal overgrowth. In contrast to the axonal role of Fos, *kay^1^* did not block the dendritic reduction caused by Wnd overexpression. The total dendritic length of MARCM clones that overexpressed Wnd in the *kay^1^* background (OE Wnd+*kay^1^*) was indistinguishable from that of Wnd-overexpressing clones (Figure 3B, B′, and C), and the number of dendrite termini was further reduced from that of Wnd-overexpressing clones. Interestingly, the *kay^1^* mutation alone caused a mild reduction in dendritic length and branch number (Figure 3B, B′, and C). This result suggests that, although Fos does not mediate the dendritic functions of the DLK pathway, it plays a minor role in supporting dendritic growth. Taken together, these results suggest that Wnd acts through Fos to specifically promote axonal growth.

### Wnd Suppresses the Expression of the Transcription Factor Knot

**Figure 4:**
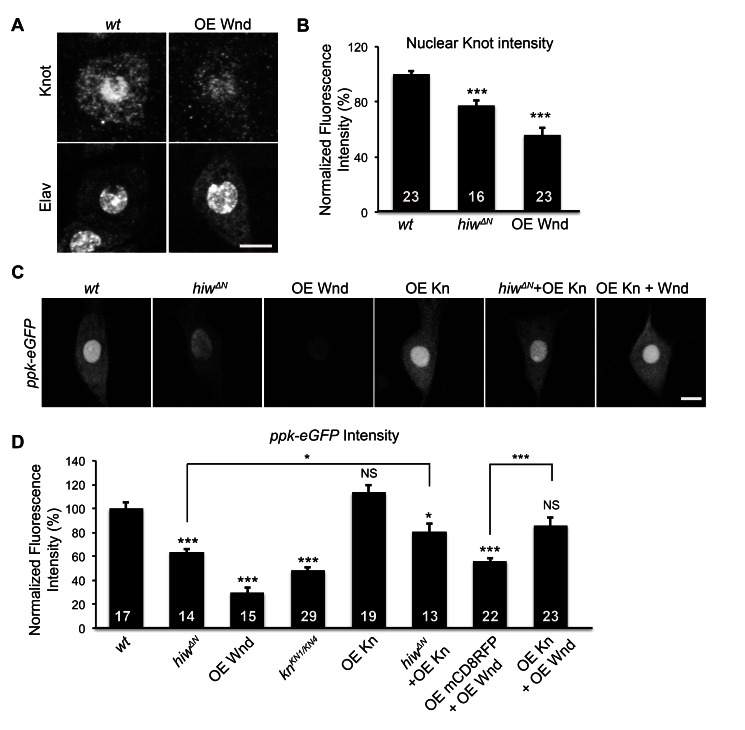
Hiw-Wnd pathway regulates the expression and transcriptional activity of the C4da-specific transcription factor Kn. (A) Overexpressing Wnd attenuates the nuclear Kn expression levels. Representative immunofluorescence images of *wt* and Wnd-overexpressing (OE Wnd) ddaC neurons labeled with antibodies against Kn (top) and Elav (bottom). Scale bar, 5 µm. (B) Quantification of the immunofluorescence intensity of nuclear Kn normalized to that of nuclear Elav in *wt*, *hiw^ΔN^*, and OE Wnd neurons. (C) Wnd overexpression down-regulates the promoter activity of the ENaC ion channel *pickpocket* (*ppk*), a known target of Kn. Representative ddaC neurons labeled with *ppk-eGFP* in neurons of the following genotypes: (1) *wt*; (2) *hiw^ΔN^*; (3) OE Wnd; (4) OE Kn; (5) *hiw^ΔN^*+OE Kn; (6) OE Kn+OE Wnd. Scale bar, 5 µm. (D) Quantification of *ppk-eGFP* fluorescent intensity in neurons of the following genotypes: (1) *wt*; (2) *hiw^ΔN^*; (3) OE Wnd; (4) *kn^KN1/KN4^*; (5) OE Kn; (6) *hiw^ΔN^*+OE Kn; (7) OE mCD8RFP+OE Kn; (8) OE Kn+OE Wnd.

In order to understand how the function of DLK pathway diverges into dendritic and axonal regulations, we hypothesized that the divergence occurred at the transcriptional level, and therefore tested the transcription factors that are known to regulate dendritic growth in da neurons. Among them, the Krüppel-like factor Dar1, the homeodomain transcription factor Cut (Ct), and zinc-finger transcription factor Knot (Kn, as known as Collier) have been shown to be essential for dendritic growth in C4da neurons. Loss-of-function mutations in each of these transcription factors severely reduce dendritic growth in C4da neurons [6],[45]–[48]. We first tested whether expression levels of these transcription factors in C4da neuron nucleus were altered in *hiw* loss-of-function mutants. No significant difference in the levels of Dar1 [6] or Cut [45] was observed between wild-type and *hiw* mutant C4da neurons (Figure S5A–C). In contrast, the nuclear levels of Kn, which belongs to the evolutionarily conserved Collier/Olf1/EBF (COE) family, were significantly reduced in both *hiw* mutant neurons and Wnd-overexpressing neurons (Figure 4A and B)

Kn is required for the expression of the ENaC ion channel Pickpocket (Ppk) in C4da neurons [46]–[48]. *Kn* loss-of-function mutations reduce *ppk* transcription [46] and suppress *ppk* promoter activity as assayed with a *ppk-eGFP* transgene (Figure 4D) [47],[48]. Furthermore, misexpression of Kn induces ectopic *ppk-eGFP* expression in neuron types that do not normally express *ppk-eGFP* [46]–[48]. Therefore, the *ppk-eGFP* transgenes may be used as readout for Kn transcriptional activity. Consistent with the reduced Kn expression by *hiw* mutations or Wnd overexpression, we found a 37% reduction in *ppk-eGFP* fluorescence intensity in the soma of *hiw* mutant C4da neurons and a 68% reduction in those of Wnd-overexpressing neurons (Figure 4C and D). Furthermore, overexpressing Kn rescued the reduced expression of *ppk-eGFP* in *hiw* mutant or Wnd-overexpressing neurons (Figure 4C and D). The correlation between *ppk*-*eGFP* fluorescence intensity and Kn levels suggests that the Hiw-Wnd pathway controls Kn transcriptional activity by regulating its protein levels. Nevertheless, it does not rule out the possibility of posttranslational regulation of Kn activity by Hiw-Wnd. Taken together, Hiw suppresses Wnd function, thus maintaining high levels of Kn protein in C4da neurons, which is required for dendritic growth.

### Knot Mediates the Hiw-Wnd Control of Dendritic Growth

**Figure 5:**
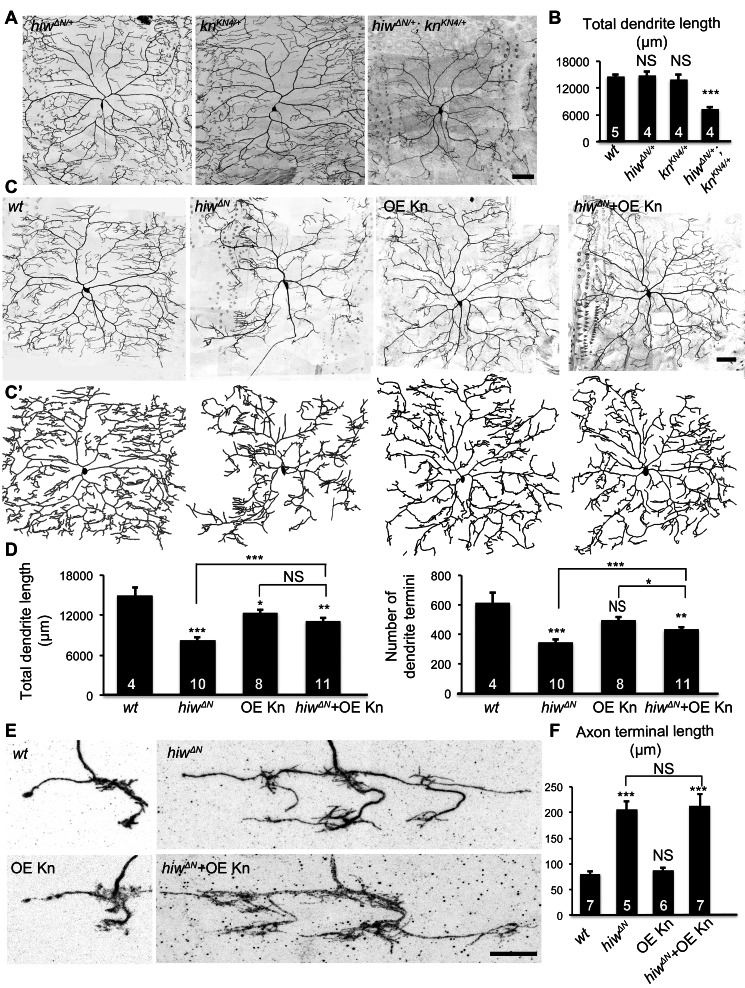
Kn specifically mediates Hiw regulation of dendritic growth. (A) *hiw* and *kn* interact genetically. Shown are representative dendrites of the following genotypes: (1) *hiw^ΔN^* heterozygote (*hiw^ΔN/+^*); (2) *kn ^KN4^* heterozygote (*kn^KN4/+^*); (3) *hiw^ΔN^* and *kn^KN4^* trans-heterozygote (*hiw^ΔN/+^*; *kn^KN4/+^*). Scale bar, 50 µm. (B) Quantification of total dendrite length of denoted genotypes. *wt* samples used for statistical analysis are the same as those in Figure 1. (C and C′) Overexpressing Kn partially rescues dendritic defects in *hiw^ΔN^* mutants. Representative dendrites (C) and tracings (C′) of ddaC MARCM clones of following genotypes: (1) *wt*; (2) *hiw^ΔN^*; (3) overexpressing Kn with MARCM (OE Kn); (4) overexpressing Knot in *hiw^ΔN^* genetic background with MARCM (*hiw^ΔN^*+OE Kn). Scale bar, 50 µm. (D) Quantification of total dendrite length (left) and number of dendrite termini (right). (E) Overexpressing Kn does not alter axon terminal morphology in *hiw^ΔN^* mutants. Shown are representative axon terminals of ddaC MARCM clones of the indicated genotypes. Scale bar, 10 µm. (F) Quantification of the length of axon terminals.

It has been demonstrated that loss-of-function mutations of *kn* cause reduction in dendritic length and branch numbers [46]–[48]. We tested potential genetic interactions between *hiw* and *kn* in controlling dendritic growth. C4da dendrites developed normally in both *hiw^ΔN/+^* heterozygous and *kn^KN4/+^* heterozygous larvae (Figure 5A and B), in which Kn expression and *ppk*-*eGFP* levels remained comparable to wild-type (Figure S5D–F). In contrast, the *hiw^ΔN/+^*; *kn^KN4/+^* transheterozygous larvae exhibited dramatically reduced dendritic growth (Figure 5A–B), revealing a strong genetic interaction between *hiw* and *kn*.

We investigated the nature of the genetic interaction by epistasis analysis. Kn overexpression resulted in a mild 16% reduction of C4da dendritic length (Figure 5C–D), possibly due to destabilized microtubules as a result of increased expression of the microtubule severing protein Spastin [6],[48]. Nevertheless, overexpressing Kn in *hiw^ΔN^* MARCM clones (*hiw^ΔN^*+OE Kn) rescued dendritic defects from 45% of reduction to 25% in dendritic length, and from 44% of reduction to 29% in dendrite termini number, as compared to wild-type (Figures 5C–D), suggesting that Kn acts downstream of Hiw to control dendrite growth.

In contrast, Kn overexpression had no effect on axonal growth in either wild-type or *hiw* mutant MARCM clones (Figure 5E–F). Taken together, our results suggest that the Hiw-Wnd pathway acts through Kn to regulate dendritic, but not axonal, growth.

### Kn Endows Neurons with the Ability to Respond to Wnd Regulation of Dendritic Growth

There are four classes of dendritic arborization (da) neurons in *Drosophila* larva, which are categorized based on the complexity of dendritic branching [49]. *Hiw* mutations elevated the expression of *puc-*lacZ [50], a reporter for Wnd activity [26], in all four classes (Figure S6A and B), suggesting that the Hiw-Wnd pathway is functional in all these neurons. However, Kn is only expressed in the class IV, and undetectable in other classes of da neurons [46]–[48]. If *hiw* acted via Kn to control dendritic growth, *hiw* mutations would not alter the dendritic morphology in class I (C1), class II (C2), and class III (C3) da neurons. Indeed, we observed that *hiw* mutant MARCM clones of C1–C3 da neurons all exhibited normal dendritic growth (Figures S7C and D, S8C and D, S9C and D), even though Hiw still restricts axonal growth in these neurons (Figures S7A and B, S8A and B, S9A and B). These observations further suggest that the Hiw-Wnd pathway regulates dendritic growth in Kn-expressing neurons.

**Figure 6:**
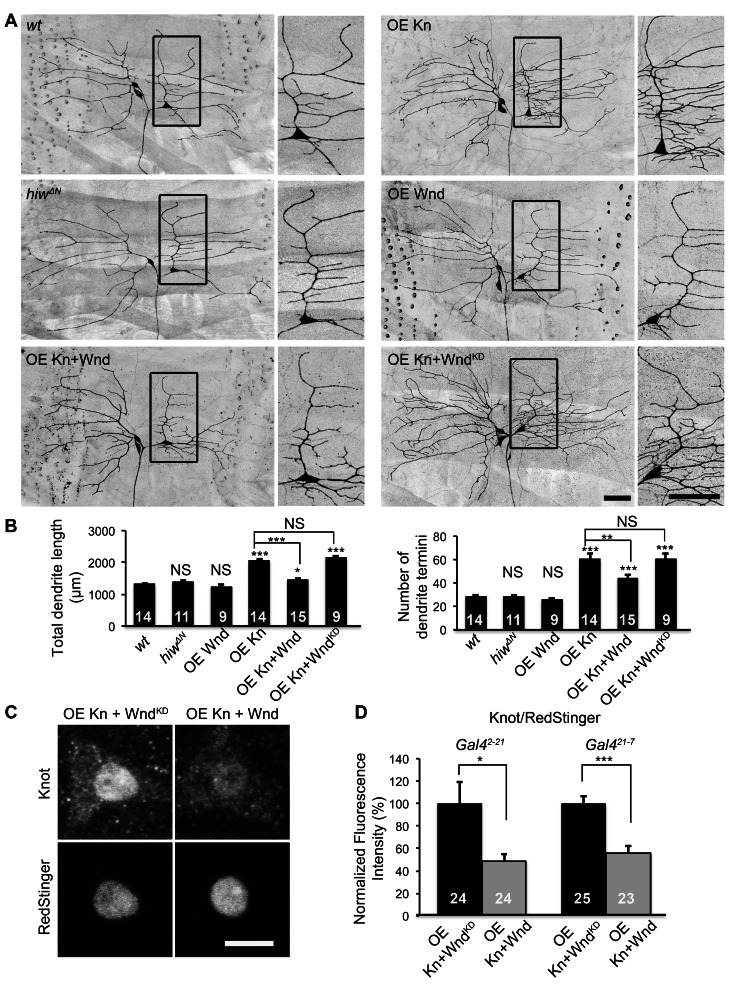
Wnd kinase inhibits dendrite growth in C1da neurons expressing ectopic Kn. (A) Wnd overexpression does not alter dendrite morphology in wild-type C1da neurons, but restrains the dendritic overgrowth caused by ectopic Kn in these neurons. Shown are representative dendrites of C1da neurons ddaD (left) and ddaE (right), labeled by *Gal4^2-21^*/*UAS-mCD8::GFP*, of the following genotypes: (1) *wt*; (2) overexpressing Kn by *Gal4^2-21^*(OE Kn); (3) *hiw^ΔN^* homozygotes (*hiw*); (4) overexpressing Wnd by *Gal4^2-21^* (OE Wnd); (5) overexpressing Kn and Wnd by *Gal4^2-21^* (OE Kn+Wnd); (6) overexpressing Kn and a kinase-dead form of Wnd by *Gal4^2-21^*(OE Kn+Wnd^KD^). Scale bar, 50 ⋯µm. Magnified views of the boxed areas are shown on the right for each genotype. (B) Quantification of total dendrite length (left) and number of dendrite termini (right) of ddaEs of denoted genotypes. (C) Wnd kinase specifically down-regulates the expression of *UAS-Kn*, but not *UAS-RedStinger* (a nuclear red fluorescent protein) [66] in a posttranscriptional manner. Representative images of ddaEs labeled with antibodies against Kn (top) and RedStinger (bottom) in “OE Kn+Wnd” and “OE Kn+Wnd^KD^” using *Gal4^2-21^*. Scale bar, 5 µm. (D) Quantification of immunofluorescence intensity of nuclear Kn normalized to that of RedStinger. Two different *Gal4* lines, *Gal4^2-21^* (left) and *Gal4^21-7^* (right), were tested in this experiment.

We next determined whether Kn expression endows neurons with the ability to respond to dendritic growth control by Wnd. Consistent with previous reports that ectopic expression of Kn in class I da (C1da) neurons leads to excessive dendritic branching and extension [47],[48], the total dendrite length was increased by 55% and the number of dendritic branches was doubled in the C1da neurons overexpressing Kn (OE Kn) compared to wild-type. Such dendritic overgrowth was considerably reduced when Wnd was overexpressed in the same neurons (Figure 6A–B), with the increase in total dendrite length inhibited from 55% to 10%. As a control, a kinase-dead form of Wnd failed to suppress Kn-induced dendritic overgrowth.

Similar to the effects in C4da neurons (Figure 4A–B), we detected a reduction of the nuclear Kn levels in C1da neurons expressing both Kn and Wnd (Figure 6C–D). It is noteworthy that, in these C1da neurons, Kn was expressed by the Gal4/UAS system, which bypasses endogenous transcriptional control. Thus, up-regulated Wnd kinase is likely to suppress Kn expression via posttranscriptional mechanism. Collectively, these results suggest that Hiw-Wnd pathway regulates dendritic growth in Kn-expressing neurons by controlling the expression of Kn.

## Discussion

In this study, we found that a single signaling pathway, consisting of the PHR E3 ubiquitin ligase Hiw and its downstream dual leucine kinase Wnd, serves not only as a negative regulator in axon growth but also as a positive regulator in dendrite growth in vivo. This is the first report, to our knowledge, to show a role for the DLK pathway in dendrite development. We further discovered that the functional divergence of this pathway is achieved through two transcription factors, Kn and Fos, which mediate the dendritic and axonal regulation, respectively.

### Three Distinct Modes of Regulations of Axonal and Dendritic Growth

**Figure 7:**
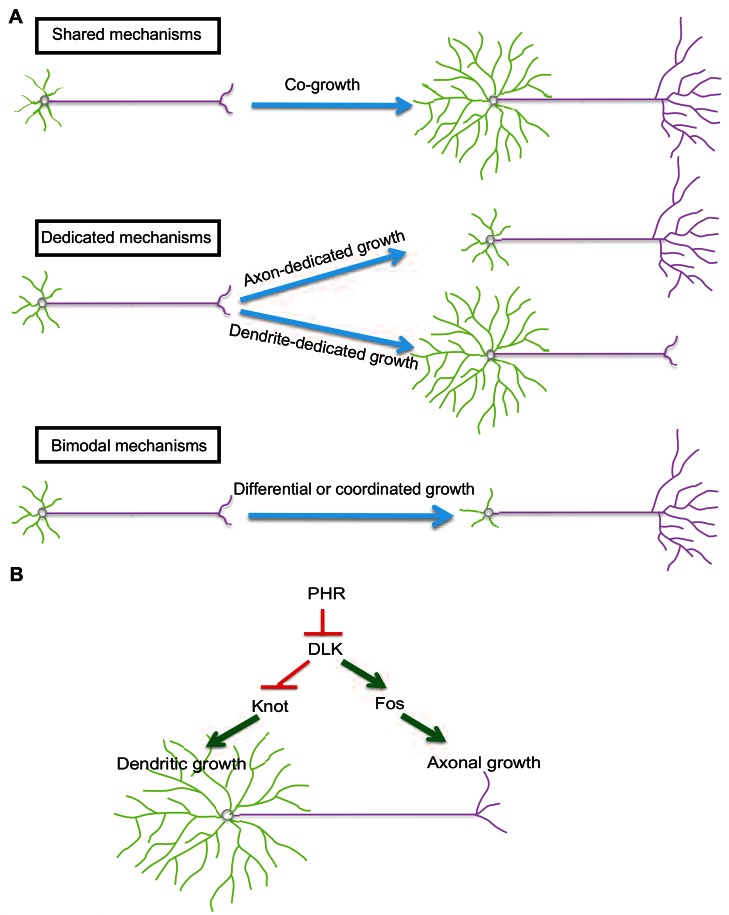
Regulatory mechanisms underlying dendritic and axonal growth. (A) Three distinct mechanisms regulating dendritic and axonal growth. Shared mechanisms control dendrite and axon co-growth. Dedicated mechanisms direct compartment-specific growth. Bimodal mechanisms differentially regulate dendritic and axonal growth. (B) A model that postulates the differential control of dendritic and axonal growth by the DLK pathway, which is based on the present study. In this model, DLK plays a dual role in neuron morphogenesis. Up-regulated DLK, caused either by *PHR* mutations or DLK overactivation, promotes the growth of axon terminals but restricts that of high-order dendritic branches. Such a dichotomous function is the result of signaling divergence into two transcriptional programs that are each dedicated to either dendritic or axonal growth. Fos serves a permissive role in the axonal regulation by DLK, whereas Kn specifically mediates the dendritic regulation by DLK.

Taking into account the current study with previous studies, three distinct modes of axonal and dendritic growth regulation have been identified: shared, dedicated, and bimodal (Figure 7A).

Shared mechanisms co-promote or co-inhibit the growth of axons and dendrites. Molecular controls that operate in shared mechanisms include cytoskeleton regulators like MAP1B (Futsch) [51], histone deacetylase HDAC6 [52],[53], and β-hexosaminidase [54].

Dedicated mechanisms provide the basis for specifically regulating the morphogenesis of only axons or only dendrites. Molecular controls at work in dedicated mechanisms can be divided into (1) axon-dedicated mechanisms, including p300 and SnoN transcription complex [2]; and (2) dendrite-dedicated mechanisms, including transcriptional factors NeuroD [3] and Dar1 [6], growth factor BMP7 [4],[5], and small GTPase Rab17 [55]. Manipulation of dedicated mechanisms leads to specific changes in the growth of either axons or dendrites, but not both. Thus, axonal growth per se does not regulate dendritic growth, and vice versa.

In contrast to dedicated mechanisms, bimodal mechanisms oppositely regulate axons and dendrites, and may serve to coordinate the growth of these separate compartments. Previous studies of different types of neuronal cultures have discovered three bimodal regulators: Sema3A [8],[9], CLASP2 [10], and Rit [11]. In this study we have identified an in vivo bimodal regulatory mechanism that involves DLK kinase. The bimodal action of the DLK signaling pathway is achieved through two “dedicated” transcriptional programs. These two programs are likely to be independent because manipulating their activities rescues either dendritic or axonal defects, but not both, in *hiw* mutants. We also observed that transgenic Hiw and Wnd were present in the axon terminals in addition to the cell body but not in dendrites (Figure S6C and D), raising the intriguing possibility that elevated Wnd function in the axon terminals might impact transcriptional activities in the cell body, and consequently influence denritic growth.

It is likely that various bimodal controls exist in different neuron types. Moreover, it is possible that these bimodal controls intersect with each other. For instance, since the actions of Sema3A are mediated through cGMP/cAMP levels [9], another bimodal regulator might also influence cGMP/cAMP levels. It will be interesting to determine whether cGMP/cAMP are involved in PHR-DLK pathway for bimodal control of dendritic and axonal growth.

### The DLK Pathway May Coordinate Dendritic and Axonal Growth After Axon Injury

Despite the requirement of DLK functions in axonal growth after axon injury [24]–[28], DLK is dispensable for axonal growth during development in the neuron types examined so far [14],[17]. Consistently, we find that loss of *dlk/wnd* does not alter either dendritic or axonal growth in *Drosophila* C4da neurons. Rather, the overabundance of DLK/Wnd caused by defective PHR/Hiw functions leads to axonal overgrowth as well as dendritic reduction. Since axon injury leads to an overabundance of DLK/Wnd function [26],[28], it is conceivable that the elevated activity of DLK/Wnd induced by axon injury not only promotes axon regeneration [24]–[28] but also restrains dendritic growth or prunes exiting dendritic branches to compensate for the increased demand of membrane or cytoskeleton supplies for axonal growth. This notion is consistent with previous studies that show dendrite retraction following axotomy in *Drosophila* da neurons [56] and mammalian cultured neurons [57],[58].

### Two Transcription Programs Directed by Kn and Fos Endow Bimodal Regulation of PHR-DLK Pathway

Although it is known that the zinc finger transcription factor Kn is essential for dendritic growth, the signaling mechanism that regulates Kn in neurons is unknown. In this study, we show that Kn specifically mediates dendritic regulation by the PHR-DLK pathway, which is supported by three lines of evidence. First, *kn* genetically interacts with *hiw* and functions downstream of *hiw* and *wnd* to regulate dendritic growth. Second, the Hiw-Wnd pathway regulates Kn expression in C4da neurons. Third, the Kn expression pattern is consistent with the presence of the Hiw-Wnd regulation of dendrite growth. Kn is selectively expressed in a subset of neurons [46]–[48],[59]. Consistent with Kn expression pattern, *hiw* mutations caused dendrite defects only in the Kn-expressing class IV neurons, and not in the other classes of da neurons that lack Kn. Interestingly, ectopic expression of Kn in class I neurons, which do not normally express Kn, is sufficient to endow the Hiw-Wnd regulation. These results strongly suggest that the PHR-DLK pathway regulates Kn to control dendrite development.

In contrast to Kn, the transcription factor Fos specifically mediates axonal regulation through Hiw-Wnd pathway. We found a two-fold role for *fos* in neuronal development. On the one hand, eliminating *fos* specifically causes dendritic reduction without affecting axon terminal length in C4da neurons. This indicates that endogenous Fos is specifically required for dendritic growth during normal development. On the other hand, the requirement of *fos* could switch to be axonal when augmented Wnd activity leads to exuberant axonal growth.

In summary, the Hiw-Wnd pathway can exert bimodal or dedicated control over dendritic and axonal growth, depending on the presence of the transcription factors that mediate its subcellular compartment-specific functions. If transcription factors for both dendritic and axonal growth are present, Hiw-Wnd signaling functions as a bimodal modulator (Figure 7B). This model provides guidance for further investigation of the molecular basis of the diversity of neuronal morphology and the differential development of dendrites and axons.

## Materials and Methods

### Fly Stocks

Fly stocks include *hiw^ΔN^* [16]; *hiw^ND8^* [16]; *UAS-Hiw::GFP* [16]; *wnd^1^* [17]; *wnd^3^* [17]; *UAS-Wnd* [17]; *UAS-Wnd^K188A^* [17]; *UAS-Wnd^KD^::GFP* [17]; *kay^1^* [60]; *ca^1^*, *FRT^82B^* [61]; *UAS-ltd::YFP* [62]; *kn^1^* [63]; *kn^KN4^* [63]; *UAS-kn* [46],[64]; *ppk-eGFP* [34]; *ppk-CD4::tdTomato* [36]; *ppk-CD4::tdGFP* [36]; *ppk-Gal4* [65]; *UAS-Kinesin::βGal* [37]; *puc-*lacZ [50]; *UAS-RedStinger* [66].

### MARCM Analyses

The MACRM analyses were performed as previously described [6]. For MARCM analyses of *hiw* mutations in four classes of da neurons, the *tubP-Gal80*, *hs-flp*, *FRT^19A^*; *Gal4^21-7^*, *UAS-mCD8::GFP* virgins were mated with males of *hiw^ΔN^,FRT^19A^*.

For MARCM analyses of *kay^1^* mutant, overexpressing Wnd, and overexpressing Wnd in *kay^1^* mutant C4da neurons, the *hs-flp*; *ppk-Gal4*, *UAS-mCD8::GFP*; *FRT^82B^ tubP-Gal80* virgins were mated with males of (1) *UAS-Wnd*; *FRT^82B^*, (2) *FRT^82B^ kay^1^*, and (3) *UAS-Wnd*; *FRT^82B^ kay^1^*, respectively.

For MARCM analyses of *ca^1^* mutations, the homozygous *FRT^82B^ ca^1^* virgins (to remove maternal contribution of wild-type Claret) were mated with males of *hs-flp*; *ppk-Gal4*, *UAS-mCD8::GFP*; *FRT^82B^ tubP-Gal80*.

To overexpress Kn in wild-type C4da neurons or in *hiw^ΔN^* mutant C4da neurons, the *tubP-Gal80*, *hs-flp*, *FRT^19A^;; ppk-Gal4*, *UAS-mCD8::GFP virgins* were mated with males of *FRT^19A^;;UAS-Kn* and *hiw^ΔN^ FRT^19A^;;UAS-Kn*, respectively.

### Immunostaining and Confocal Imaging

Embryos and third instar larvae were dissected and immunostained as previously described [7]. The following primary antibodies were used: mouse anti-GFP (Invitrogen, 1∶2,000), chick anti-GFP (1∶2,000), rabbit anti-RFP (Rockland, 1∶2,000), guinea pig anti-Knot (gift from Adrian Moore, 1∶1,000), rat anti-Elav (DSHB, 1∶500), guinea pig anti-Dar1 (1∶1,000) [6], rabbit anti-Cut (1∶1,000) [67], rabbit anti-βGAL (Cappel, 1∶5,000), and mouse anti-βGAL (DSHB, 1∶100).

Confocal imaging was performed with a Leica SP5 confocal system. Only da neurons from abdominal segment 4 to 6 were imaged for quantification of dendrites and axons to ensure consistency.

To compare protein expression levels in C4da neurons, larvae of different genotypes in the same experimental group were processed simultaneously. The same setting for image acquisition was applied to the same experimental group and signal saturation was minimized. Fluorescence intensities of different genotypes were normalized to wild-type (Figures 4 and S5) or the OE Wnd^KD^ control group (Figure 6).

### Quantifications and Statistical Analysis

To quantify protein levels, mean fluorescence intensity of the region of interest in each channel was measured with NIH ImageJ software. For axon terminal and dendritic morphology, manual tracing was conducted with Neurolucida software. Branches shorter than 5 µm were excluded. For consistency, da neurons located between segment A4 and A6 from size-matched third instar larvae were imaged and analyzed in all experiments.

In all of the bar charts of quantification, the numbers in the bars indicate the sample numbers. Values and error bars indicate mean ± SEM. Two-tailed unpaired student *t*-test was used. *p* values were indicated as: not significant (NS) *p*>0.05, * *p*<0.05, ** *p*<0.01, *** *p*<0.001.

## Supporting Information

Figure S1 Hiw-Wnd signaling pathway operates in C4da neurons to regulate axon terminal growth. (A) A schematic of the C4da neuron system in *Drosophila* larvae. The cell bodies of the three C4da neurons—ddaC (green), v'ada (yellow), and vdaB (red)—are located from dorsal to ventral, a pattern that is repeated in each hemi-segment. The dendrites of these three neurons tile the body wall, and their axons (blue) fasciculate to enter the VNC. The C4da axon terminals form a ladder-like structure in VNC. (A′) Illustrations of representative axon terminals of individual ddaC, v'ada, and vdaB (top) and their arrangement in the C4da neuropil (blue) (bottom). (B) *hiw* mutations induce axon overgrowth in C4da neurons. Shown are representative images of C4da neuropil between segment A4 and A6 of wild-type (*wt*) and *hiw^ΔN^* homozygotes (*hiw*). (C) Loss of *wnd* blocks axonal overgrowth in *hiw* mutants, and Wnd overexpression induces axon overgrowth. Shown are representative images of C4da neuropil between segment A4 and A6 of the following genotypes: (1) *wnd^1^/wnd^3^* (*wnd*); (2) *hiw^ΔN^*; *wnd^1^/wnd^3^* double mutants (*hiw*; *wnd*); (3) Wnd overexpression by *ppkGal4* (OE Wnd); (4) overexpression of a kinase dead form (K188A) of Wnd by *ppkGal4* (OE Wnd^KD)^. (B–C) The magnified views of boxed area between A5 and A6 are shown on the right of each genotype. Scale bar, 5 µm. (D) Percentage of hemi-segments with more than three connectives between A5 and A6.
(TIF)

Figure S2 Hiw is dispensable for axon specification and early axon and dendrite development. (A) Loss of *hiw* does not alter axon or dendrite identity. Axon-specific marker Kinesin-β-galactosidase (Magenta) exclusively localizes to the axons of C4da neurons labeled by *ppk-CD4::tdGFP* (green) in *wt* and *hiw^ΔN^* larvae. Scale bar, 20 µm. (B) Loss of *hiw* does not affect axon pathfinding into the VNC (left panels) or early dendritic extension in stage 16 embryos (right panels). These results were collected from embryos devoid of both maternal and zygotic *hiw* functions. Scale bar, 10 µm.
(TIF)

Figure S3 Hiw is required cell-autonomously for dendritic and axonal growth in C4da neurons. (A–B) Overexpressing Hiw exclusively in C4da neurons does not alter axon terminal growth or dendritic growth, but restores the axonal and dendritic defects in *hiw^ΔN^* mutants. Shown are representative A4–A6 neuropils (A) and ddaCs dendrites (B) of following genotypes: (1) overexpressing Hiw by *ppkGal4* (OE Hiw); (2) overexpressing Hiw by *ppkGal4* in *hiw^ΔN^* homozygous mutants (*hiw^ΔN^*+OE Hiw). Scale bar in (A), 10 µm. Scale bar in (B), 50 µm. (C) Bar charts showing the percentage of hemi-segments with more than three connectives between A5 and A6 (left), total dendrite length (middle), and number of dendrite termini (right). Samples of *wt* and *hiw^ΔN^* that are used for statistical analysis are the same as those in Figure 1.
(TIF)

Figure S4 The Ca-Ltd trafficking pathway is dispensable for dendritic and axonal growth. (A–D) *Claret* (*ca*) is not required for either axonal or dendritic growth. Representative axon terminals (A) and dendrites (C) of ddaC MARCM clones in *wt* (*FRT^82B^*) and *ca^1^* (*ca^1^*, *FRT^82B^*) are shown. Maternal contribution of *ca* was removed by using homozygous *ca^1^*, *FRT^82B^* mutant females in the MARCM cross. (B and D) Quantification of axon terminal length (B), total dendrite length (D, top), and number of dendrite termini (D, bottom) of *wt* and *ca^1^* MARCM clones. Samples of *wt* used for statistical analysis are the same as those in Figure 3. (E–H) Overexpressing Ltd fails to rescue axon or dendrite defects in *hiw^ΔN^* mutants. Shown are representative A4–A6 neuropils (E) and ddaCs dendrites (G) of the following genotypes: overexpressing Ltd by *ppkGal4* (OE Ltd), and overexpressing Ltd by *ppkGal4* in *hiw^ΔN^* homozygotes genetic background (*hiw^ΔN^*+OE Ltd). (F and H) Bar charts showing the percentage of hemi-segments with more than three connectives between A5 and A6 (F), total dendrite length (H, top), and number of dendrite termini (H, bottom). Samples of *wt* and *hiw^ΔN^* that are used for statistical analysis are the same as those in Figures 1 and S1. Scale bar in (A and E), 10 µm. Scale bar in (C and G), 50 µm.
(TIF)

Figure S5 Normal Dar1 and Cut expression in *hiw* mutants and unaltered Kn levels in *kn* heterozygotes. (A) Dar1 nuclear expression levels are comparable between *wt* and *hiw^ΔN^* mutants. Shown are representative immunofluorescence of ddaC neurons stained with antibodies against Dar1 (top) and Elav (bottom). (B) Cut nuclear expression levels are comparable between *wt* and *hiw^ΔN^* mutants. Shown are representative immunofluorescence of ddaC neurons stained with antibodies against Cut (top) and Elav (bottom). (C) Quantification of nuclear immunofluorescence intensity of Dar1 (left) or Cut (right) normalized to nuclear Elav immunofluorescence intensity. (D) Knot nuclear expression levels are unaltered in *kn^KN4/+^*. Shown are representative immunofluorescence of ddaC neurons stained with antibodies against Knot (top) and Elav (bottom). (E) *ppk-eGFP* levels are unaltered in *kn^KN4/+^*. Representative ddaC neurons labeled with *ppk-eGFP* in *wt* and *kn^KN4/+^*. (F) Quantification of nuclear immunofluorescence intensity of Knot normalized to nuclear Elav immunofluorescence intensity (left) and *ppk-eGFP* fluorescent intensity (right). Scale bar, 5 µm.
(TIF)

Figure S6 The expression pattern of Hiw and Wnd in da neurons. (A–B) Hiw functions in all four classes of da neurons. (A) The expression of *puc*-lacZ, a reporter for Wnd activity, is elevated by *hiw* mutations in class I–IV da neurons. Shown are representative immunofluorescence of C1da (ddaE), C2da (ddaB), C3da (ddaF), and C4da (ddaC) neurons stained with an anti-βGal antibody. Scale bar, 5 µm. (B) Quantification of nuclear immunofluorescence of β-Gal expressed by *puc*-lacZ. (C–D) Hiw and Wnd are localized to the soma and axon terminals but not the dendrites of C4da neurons. (C) Localization of mCD8::RFP (top) and Hiw::GFP (bottom) in the cell body (left), axon terminals (middle), and dendrites (right) of ddaC neurons that overexpress Hiw:GFP in *hiw^ΔN^* homozygous mutants. (D) Localization of mCD8::RFP (top) and Wnd^KD^::GFP (bottom) in the cell body (left), axon terminals (middle), and dendrites (right) of ddaC neurons. Scale bar in (C) and (D), 10 µm.
(TIF)

Figure S7 Hiw specifically restrains axonal growth in class I da neurons in a cell-autonomous manner. (A–B) Loss of *hiw* causes axonal overgrowth in class I da (C1da) neurons. (A) Representative axon terminals of MARCM clones of the C1da neurons ddaD and ddaE are shown. Open arrowheads indicate where the axon enters the sensory neuropil. Scale bar, 10 µm. (B) Quantification of axon terminal length of *wt* and *hiw^ΔN^* MARCM clones. (C–D) Loss of *hiw* does not alter dendritic growth in C1da neurons. Representative dendrites of MARCM clones of ddaD and ddaE are shown. (D) Quantification of total dendrite length (top) and number of dendrite termini (bottom) of *wt* and *hiw^ΔN^* MARCM clones. Scale bar, 50 µm.
(TIF)

Figure S8 Hiw specifically restrains axon growth in class II da neurons in a cell-autonomous manner. (A–B) Loss of *hiw* causes axonal overgrowth in class II da (C2da) neurons. (A) Representative axon terminals of MARCM clones of the C2da neurons ddaB are shown. Open arrowheads indicate where the axon enters the sensory neuropil. Scale bar, 10 µm. (B) Quantification of axon terminal length of *wt* and *hiw^ΔN^* MARCM clones. (C–D) Loss of *hiw* does not alter dendritic growth in C2da neurons. Representative dendrites of MARCM clones of ddaB are shown. (D) Quantification of total dendrite length (top) and number of dendrite termini (bottom) of *wt* and *hiw^ΔN^* MARCM clones. Scale bar, 50 µm.
(TIF)

Figure S9 Hiw specifically restrains axon growth in class III da neurons in a cell-autonomous manner. (A–B) Loss of *hiw* causes axonal overgrowth in class III da (C3da) neurons. (A) Representative axon terminals of MARCM clones of the C3da neurons ddaF are shown. Open arrowheads indicate where the axon enters the sensory neuropil. Scale bar, 10 µm. (B) Quantification of axon terminal length of *wt* and *hiw^ΔN^* MARCM clones. (C–D) Loss of *hiw* does not alter dendritic growth in C3da neurons. Representative dendrites of MARCM clones of ddaF are shown. (D) Quantification of total dendrite length (top) and number of dendritic spikes (bottom) of *wt* and *hiw^ΔN^* MARCM clones. Scale bar, 50 µm.
(TIF)

## Abbreviations

β-Gal: β-galactosidase
C4da: class IV dendritic arborization
*ca*: *claret*
*ltd*: *lightoid*
da: dendritic arborization
DLK: dual leucine zipper kinase
*hiw*: *highwire*
*kn*: *knot*
PHR: Pam/Highwire/RPM-1
*ppk*: *pickpocket*
VNC: ventral nerve cord
*wnd*: *wallenda*
