# Supplementary figures and images for: Bimodal Control of Dendritic and Axonal Growth by the Dual Leucine Zipper Kinase Pathway
Source: PLoS Biol. 2013 Jun 4;11(6):e1001572. doi: 10.1371/journal.pbio.1001572 (PMC3672216; doi:10.1371/journal.pbio.1001572)

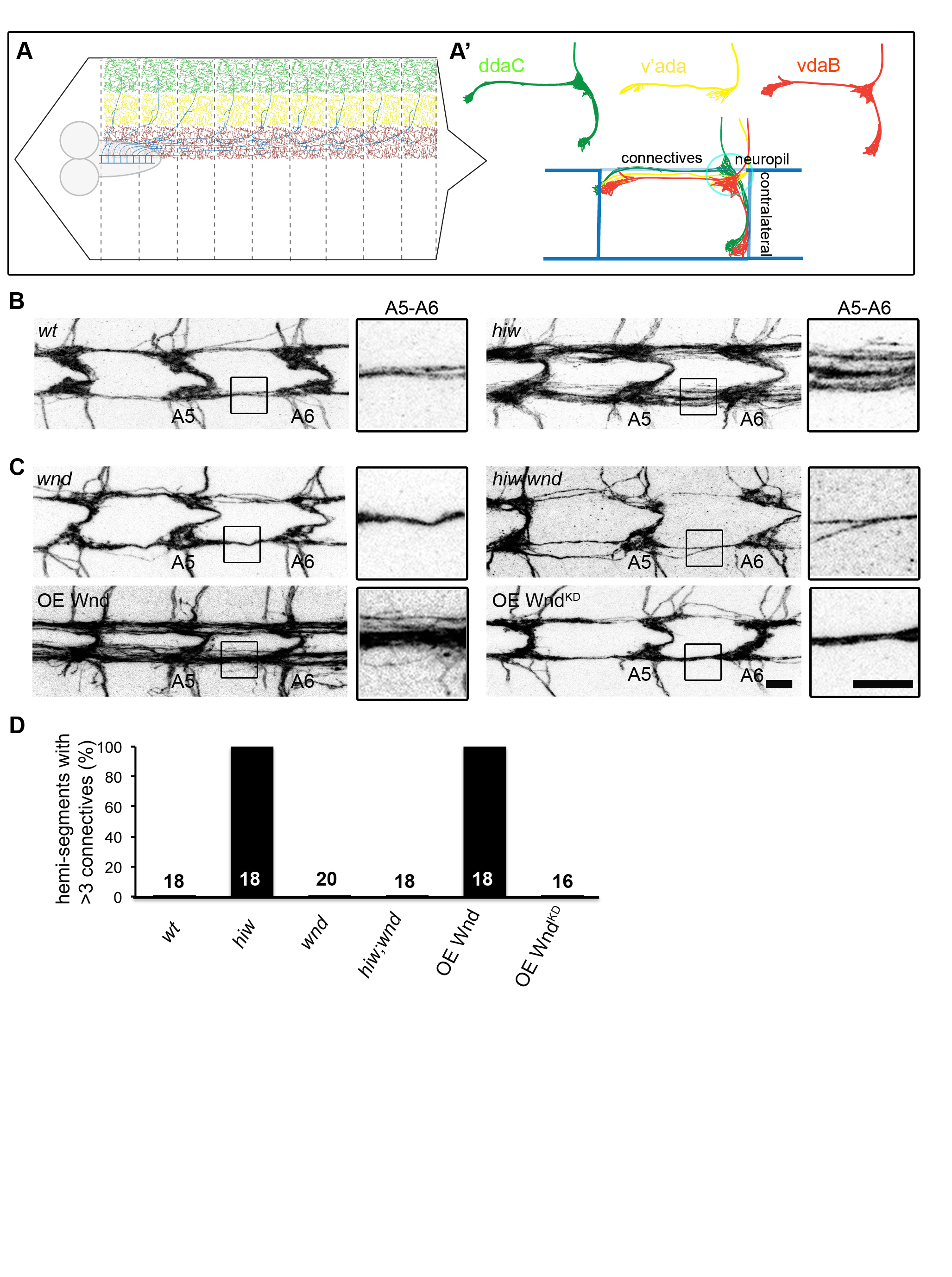

Supplement: Figure S1 — Hiw-Wnd signaling pathway operates in C4da neurons to regulate axon terminal growth. (A) A schematic of the C4da neuron system in Drosophila larvae. The cell bodies of the three C4da neurons—ddaC (green), v'ada (yellow), and vdaB (red)—are located from dorsal to ventral, a pattern that is repeated in each hemi-segment. The dendrites of these three neurons tile the body wall, and their axons (blue) fasciculate to enter the VNC. The C4da axon terminals form a ladder-like structure in VNC. (A′) Illustrations of representative axon terminals of individual ddaC, v'ada, and vdaB (top) and their arrangement in the C4da neuropil (blue) (bottom). (B) hiw mutations induce axon overgrowth in C4da neurons. Shown are representative images of C4da neuropil between segment A4 and A6 of wild-type (wt) and hiwΔN homozygotes (hiw). (C) Loss of wnd blocks axonal overgrowth in hiw mutants, and Wnd overexpression induces axon overgrowth. Shown are representative images of C4da neuropil between segment A4 and A6 of the following genotypes: (1) wnd1/wnd3 (wnd); (2) hiwΔN; wnd1/wnd3 double mutants (hiw; wnd); (3) Wnd overexpression by ppkGal4 (OE Wnd); (4) overexpression of a kinase dead form (K188A) of Wnd by ppkGal4 (OE WndKD). (B–C) The magnified views of boxed area between A5 and A6 are shown on the right of each genotype. Scale bar, 5 µm. (D) Percentage of hemi-segments with more than three connectives between A5 and A6. (TIF) [file pbio.1001572.s001.tif]

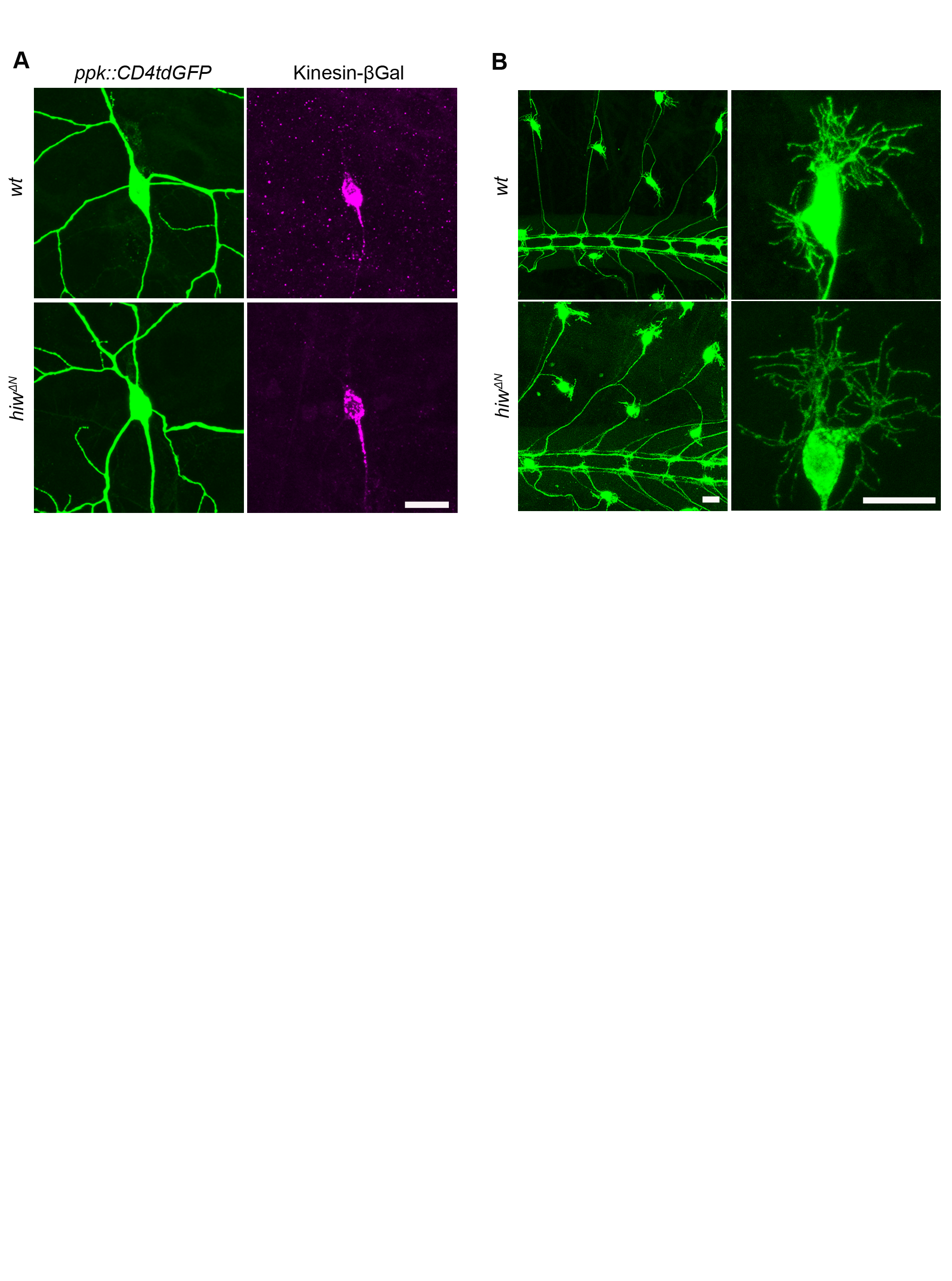

Supplement: Figure S2 — Hiw is dispensable for axon specification and early axon and dendrite development. (A) Loss of hiw does not alter axon or dendrite identity. Axon-specific marker Kinesin-β-galactosidase (Magenta) exclusively localizes to the axons of C4da neurons labeled by ppk-CD4::tdGFP (green) in wt and hiwΔN larvae. Scale bar, 20 µm. (B) Loss of hiw does not affect axon pathfinding into the VNC (left panels) or early dendritic extension in stage 16 embryos (right panels). These results were collected from embryos devoid of both maternal and zygotic hiw functions. Scale bar, 10 µm. (TIF) [file pbio.1001572.s002.tif]

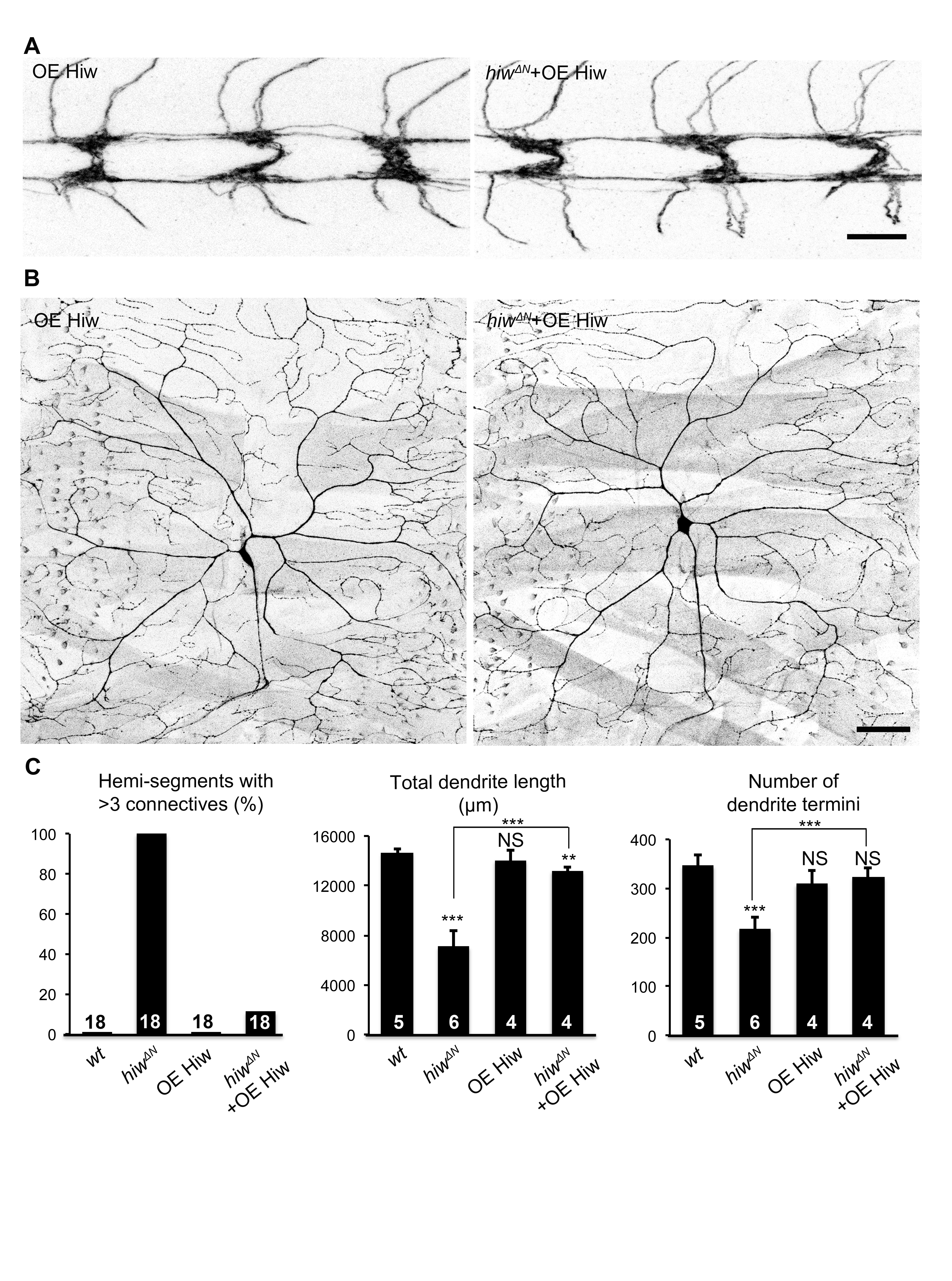

Supplement: Figure S3 — Hiw is required cell-autonomously for dendritic and axonal growth in C4da neurons. (A–B) Overexpressing Hiw exclusively in C4da neurons does not alter axon terminal growth or dendritic growth, but restores the axonal and dendritic defects in hiwΔN mutants. Shown are representative A4–A6 neuropils (A) and ddaCs dendrites (B) of following genotypes: (1) overexpressing Hiw by ppkGal4 (OE Hiw); (2) overexpressing Hiw by ppkGal4 in hiwΔN homozygous mutants (hiwΔN+OE Hiw). Scale bar in (A), 10 µm. Scale bar in (B), 50 µm. (C) Bar charts showing the percentage of hemi-segments with more than three connectives between A5 and A6 (left), total dendrite length (middle), and number of dendrite termini (right). Samples of wt and hiwΔN that are used for statistical analysis are the same as those in Figure 1. (TIF) [file pbio.1001572.s003.tif]

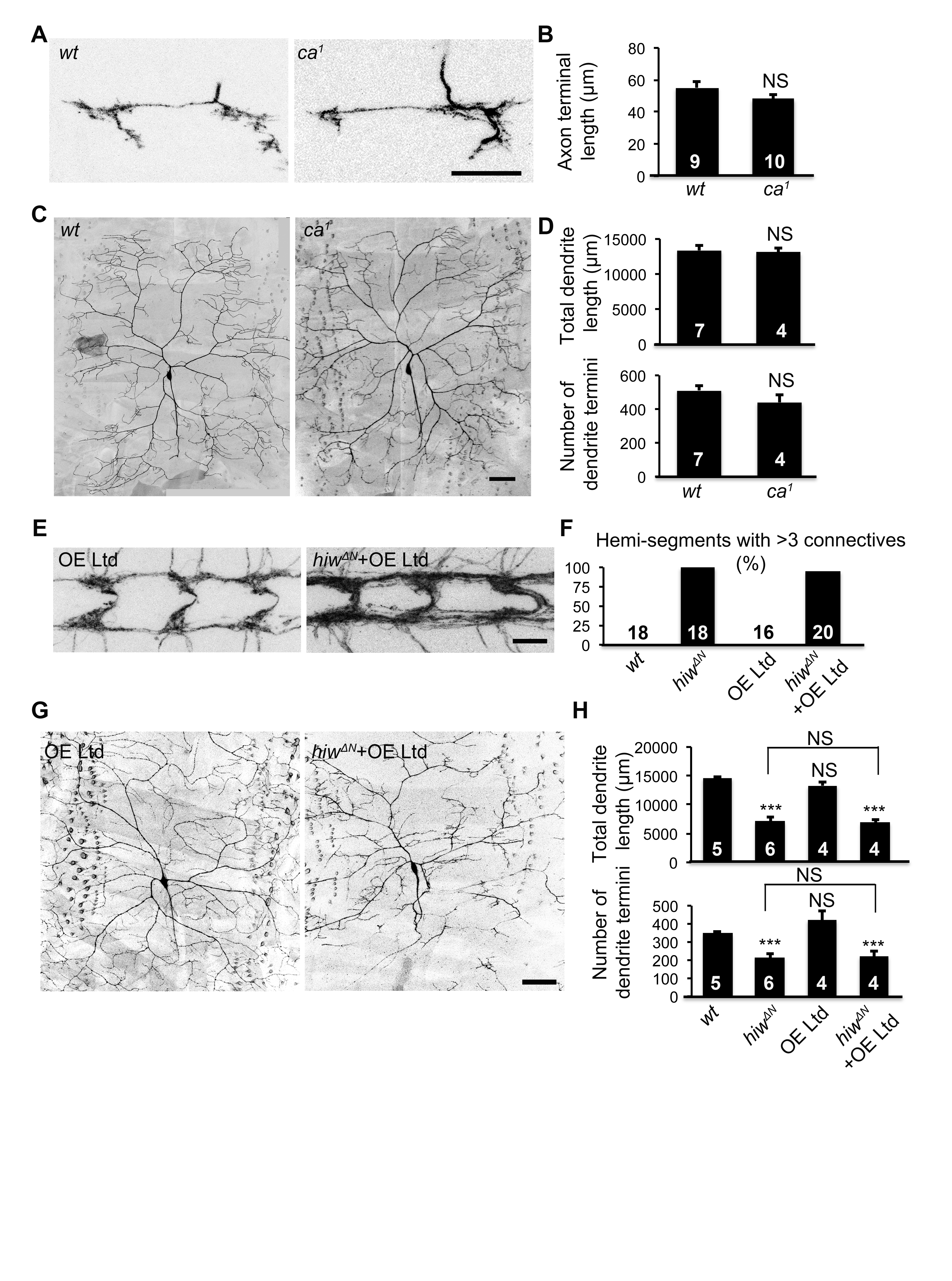

Supplement: Figure S4 — The Ca-Ltd trafficking pathway is dispensable for dendritic and axonal growth. (A–D) Claret (ca) is not required for either axonal or dendritic growth. Representative axon terminals (A) and dendrites (C) of ddaC MARCM clones in wt (FRT82B) and ca1 (ca1, FRT82B) are shown. Maternal contribution of ca was removed by using homozygous ca1, FRT82B mutant females in the MARCM cross. (B and D) Quantification of axon terminal length (B), total dendrite length (D, top), and number of dendrite termini (D, bottom) of wt and ca1 MARCM clones. Samples of wt used for statistical analysis are the same as those in Figure 3. (E–H) Overexpressing Ltd fails to rescue axon or dendrite defects in hiwΔN mutants. Shown are representative A4–A6 neuropils (E) and ddaCs dendrites (G) of the following genotypes: overexpressing Ltd by ppkGal4 (OE Ltd), and overexpressing Ltd by ppkGal4 in hiwΔN homozygotes genetic background (hiwΔN+OE Ltd). (F and H) Bar charts showing the percentage of hemi-segments with more than three connectives between A5 and A6 (F), total dendrite length (H, top), and number of dendrite termini (H, bottom). Samples of wt and hiwΔN that are used for statistical analysis are the same as those in Figures 1 and S1. Scale bar in (A and E), 10 µm. Scale bar in (C and G), 50 µm. (TIF) [file pbio.1001572.s004.tif]

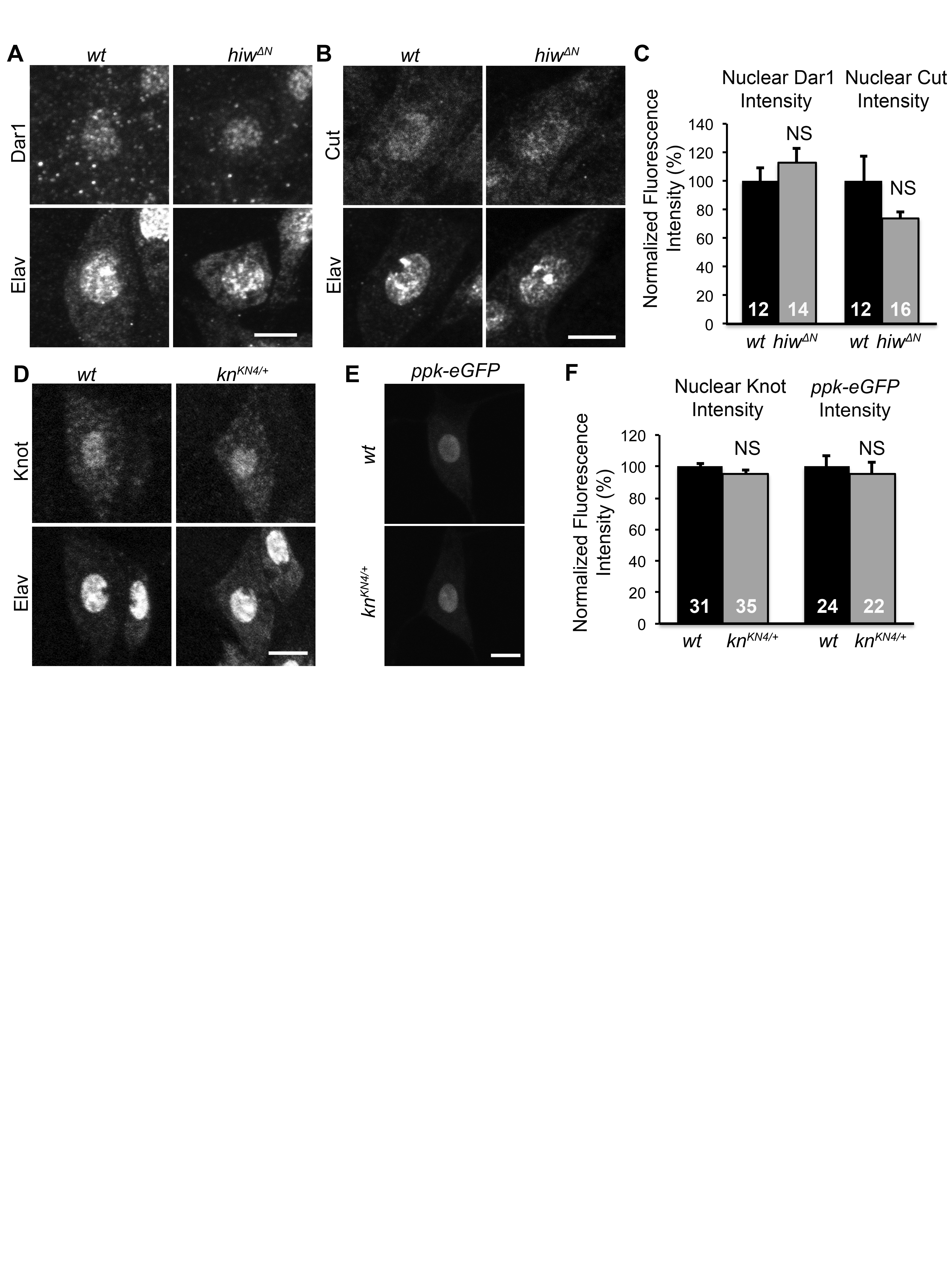

Supplement: Figure S5 — Normal Dar1 and Cut expression in hiw mutants and unaltered Kn levels in kn heterozygotes. (A) Dar1 nuclear expression levels are comparable between wt and hiwΔN mutants. Shown are representative immunofluorescence of ddaC neurons stained with antibodies against Dar1 (top) and Elav (bottom). (B) Cut nuclear expression levels are comparable between wt and hiwΔN mutants. Shown are representative immunofluorescence of ddaC neurons stained with antibodies against Cut (top) and Elav (bottom). (C) Quantification of nuclear immunofluorescence intensity of Dar1 (left) or Cut (right) normalized to nuclear Elav immunofluorescence intensity. (D) Knot nuclear expression levels are unaltered in knKN4/+. Shown are representative immunofluorescence of ddaC neurons stained with antibodies against Knot (top) and Elav (bottom). (E) ppk-eGFP levels are unaltered in knKN4/+. Representative ddaC neurons labeled with ppk-eGFP in wt and knKN4/+. (F) Quantification of nuclear immunofluorescence intensity of Knot normalized to nuclear Elav immunofluorescence intensity (left) and ppk-eGFP fluorescent intensity (right). Scale bar, 5 µm. (TIF) [file pbio.1001572.s005.tif]

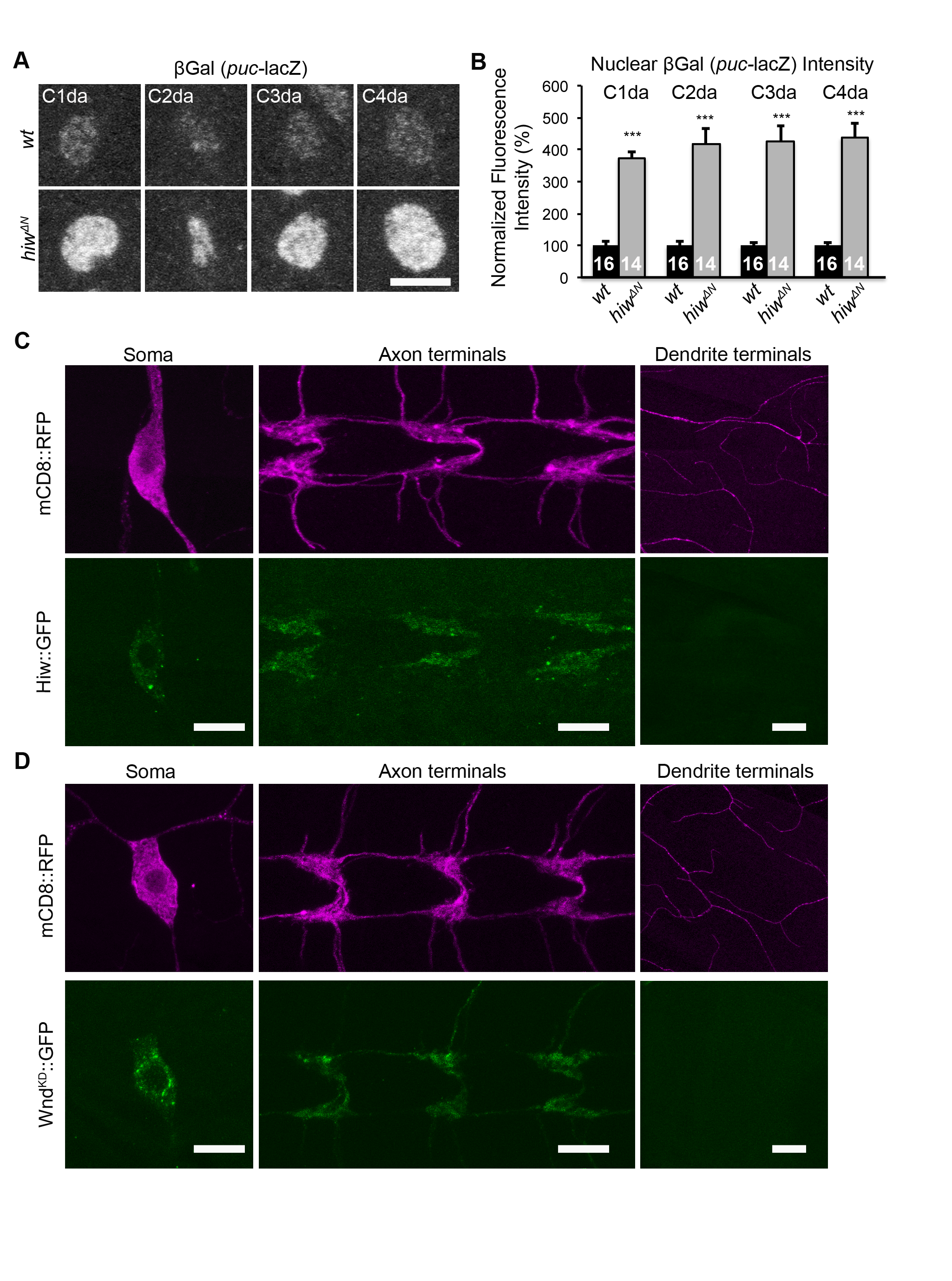

Supplement: Figure S6 — The expression pattern of Hiw and Wnd in da neurons. (A–B) Hiw functions in all four classes of da neurons. (A) The expression of puc-lacZ, a reporter for Wnd activity, is elevated by hiw mutations in class I–IV da neurons. Shown are representative immunofluorescence of C1da (ddaE), C2da (ddaB), C3da (ddaF), and C4da (ddaC) neurons stained with an anti-βGal antibody. Scale bar, 5 µm. (B) Quantification of nuclear immunofluorescence of β-Gal expressed by puc-lacZ. (C–D) Hiw and Wnd are localized to the soma and axon terminals but not the dendrites of C4da neurons. (C) Localization of mCD8::RFP (top) and Hiw::GFP (bottom) in the cell body (left), axon terminals (middle), and dendrites (right) of ddaC neurons that overexpress Hiw:GFP in hiwΔN homozygous mutants. (D) Localization of mCD8::RFP (top) and WndKD::GFP (bottom) in the cell body (left), axon terminals (middle), and dendrites (right) of ddaC neurons. Scale bar in (C) and (D), 10 µm. (TIF) [file pbio.1001572.s006.tif]

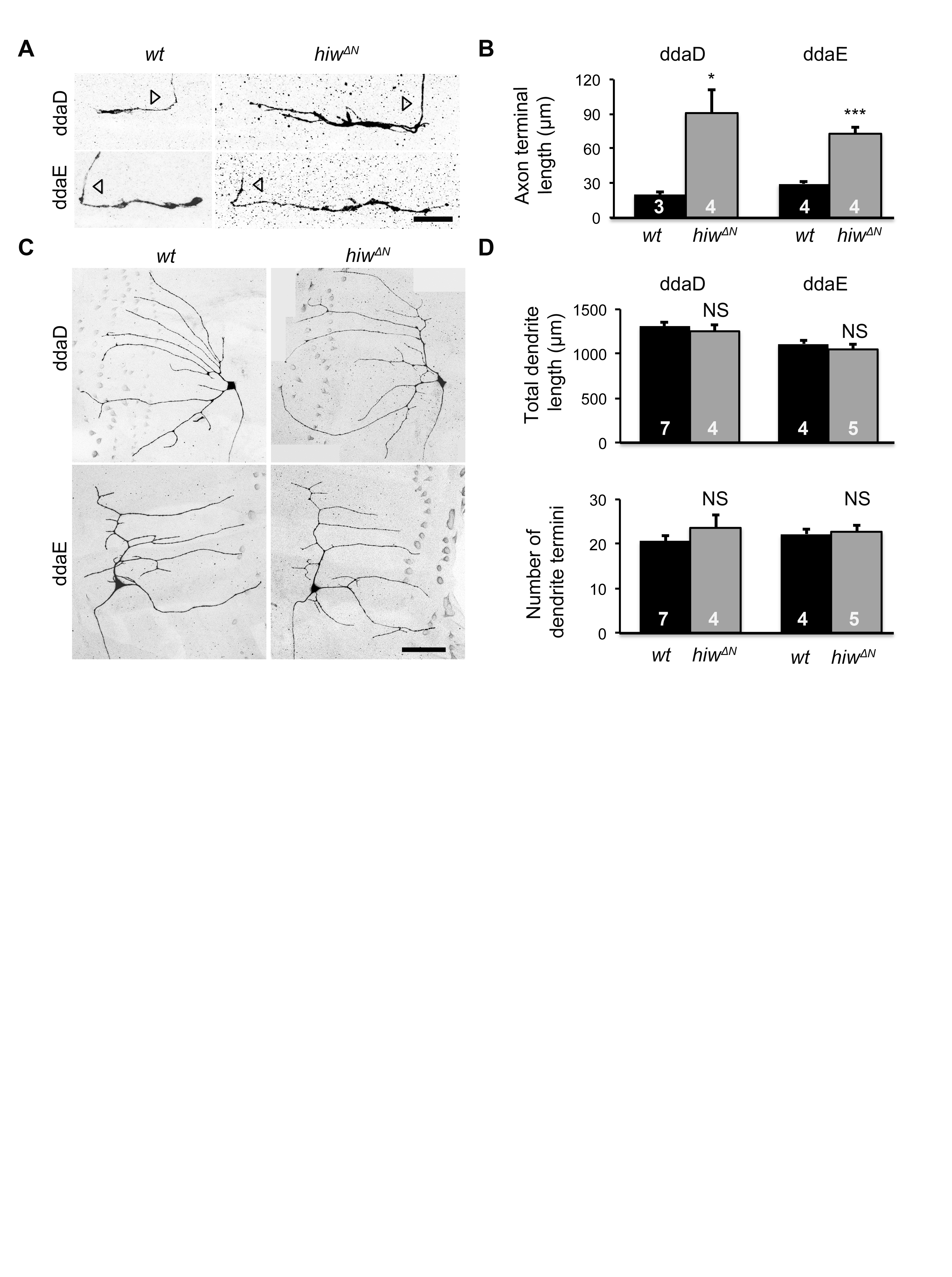

Supplement: Figure S7 — Hiw specifically restrains axonal growth in class I da neurons in a cell-autonomous manner. (A–B) Loss of hiw causes axonal overgrowth in class I da (C1da) neurons. (A) Representative axon terminals of MARCM clones of the C1da neurons ddaD and ddaE are shown. Open arrowheads indicate where the axon enters the sensory neuropil. Scale bar, 10 µm. (B) Quantification of axon terminal length of wt and hiwΔN MARCM clones. (C–D) Loss of hiw does not alter dendritic growth in C1da neurons. Representative dendrites of MARCM clones of ddaD and ddaE are shown. (D) Quantification of total dendrite length (top) and number of dendrite termini (bottom) of wt and hiwΔN MARCM clones. Scale bar, 50 µm. (TIF) [file pbio.1001572.s007.tif]

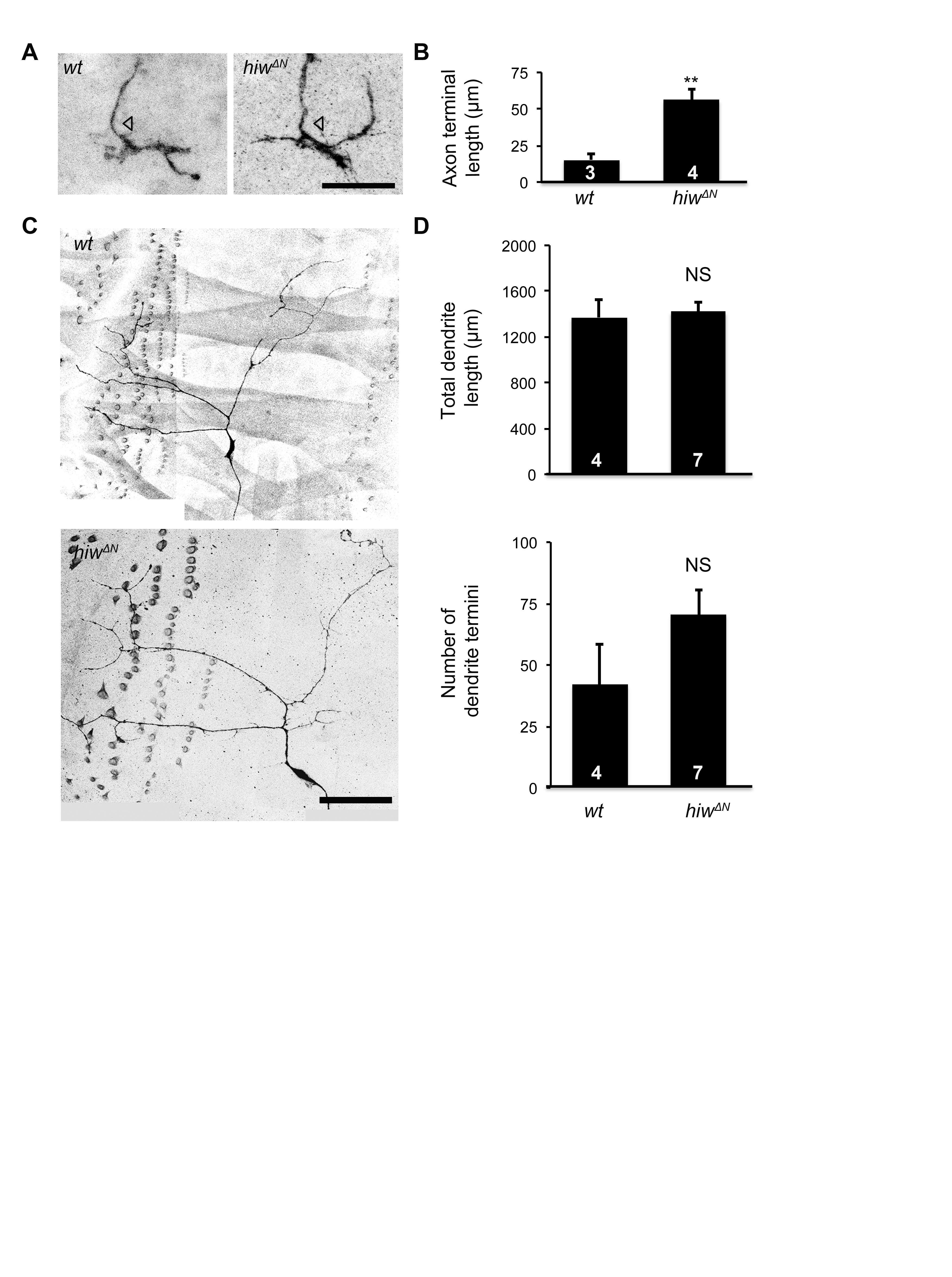

Supplement: Figure S8 — Hiw specifically restrains axon growth in class II da neurons in a cell-autonomous manner. (A–B) Loss of hiw causes axonal overgrowth in class II da (C2da) neurons. (A) Representative axon terminals of MARCM clones of the C2da neurons ddaB are shown. Open arrowheads indicate where the axon enters the sensory neuropil. Scale bar, 10 µm. (B) Quantification of axon terminal length of wt and hiwΔN MARCM clones. (C–D) Loss of hiw does not alter dendritic growth in C2da neurons. Representative dendrites of MARCM clones of ddaB are shown. (D) Quantification of total dendrite length (top) and number of dendrite termini (bottom) of wt and hiwΔN MARCM clones. Scale bar, 50 µm. (TIF) [file pbio.1001572.s008.tif]

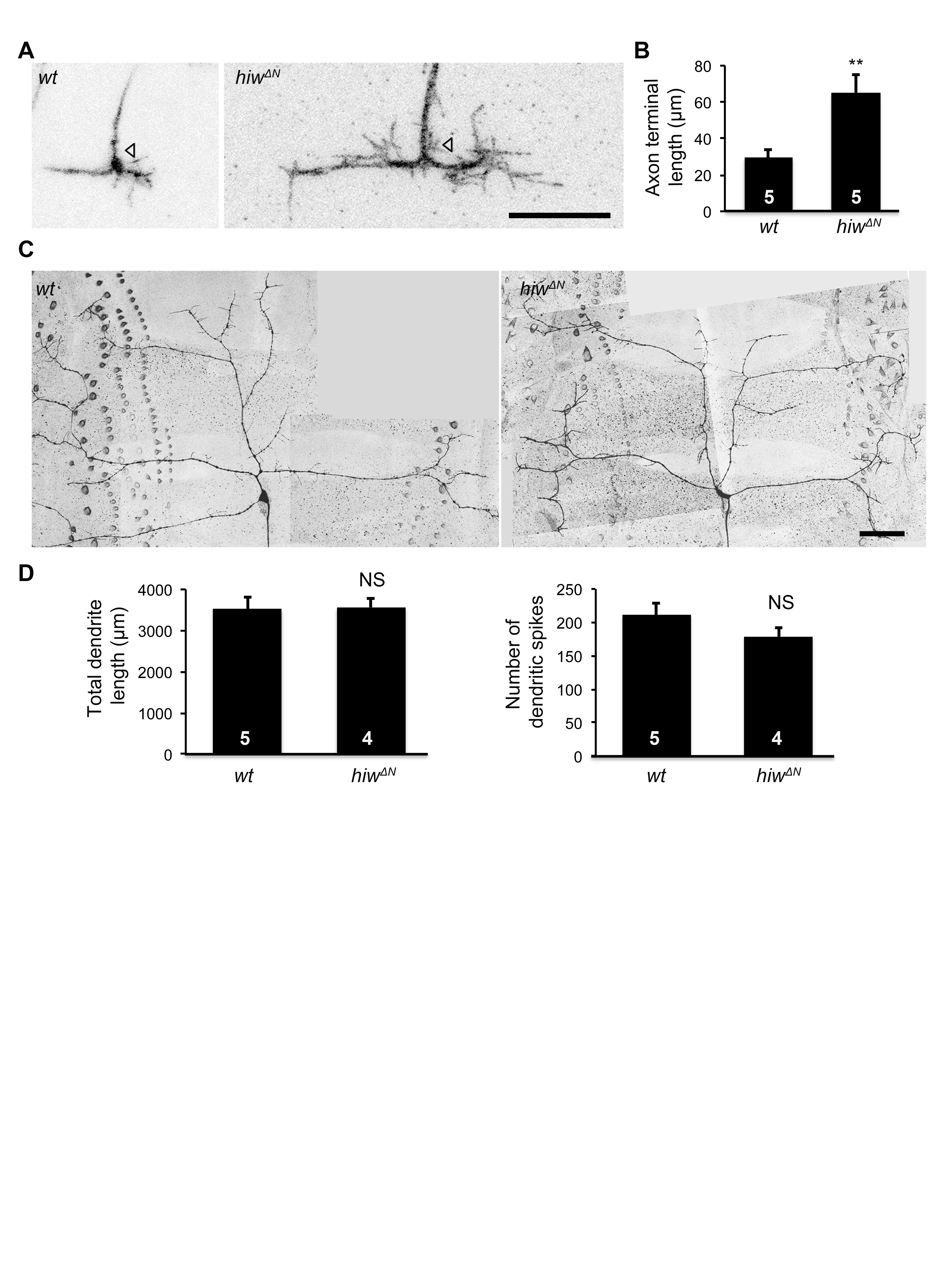

Supplement: Figure S9 — Hiw specifically restrains axon growth in class III da neurons in a cell-autonomous manner. (A–B) Loss of hiw causes axonal overgrowth in class III da (C3da) neurons. (A) Representative axon terminals of MARCM clones of the C3da neurons ddaF are shown. Open arrowheads indicate where the axon enters the sensory neuropil. Scale bar, 10 µm. (B) Quantification of axon terminal length of wt and hiwΔN MARCM clones. (C–D) Loss of hiw does not alter dendritic growth in C3da neurons. Representative dendrites of MARCM clones of ddaF are shown. (D) Quantification of total dendrite length (top) and number of dendritic spikes (bottom) of wt and hiwΔN MARCM clones. Scale bar, 50 µm. (TIF) [file pbio.1001572.s009.tif]
